# Drought Stress Predominantly Endures *Arabidopsis thaliana* to *Pseudomonas syringae* Infection

**DOI:** 10.3389/fpls.2016.00808

**Published:** 2016-06-07

**Authors:** Aarti Gupta, Sandeep K. Dixit, Muthappa Senthil-Kumar

**Affiliations:** National Institute of Plant Genome ResearchNew Delhi, India

**Keywords:** combined stress protocol, individual stress, combined stress, tailored responses, stress-interaction, stress intensity

## Abstract

Plant responses to a combination of drought and bacterial pathogen infection, an agronomically important and altogether a new stress, are not well-studied. While occurring concurrently, these two stresses can lead to synergistic or antagonistic effects on plants due to stress-interaction. It is reported that plant responses to the stress combinations consist of both strategies, unique to combined stress and those shared between combined and individual stresses. However, the combined stress response mechanisms governing stress interaction and net impact are largely unknown. In order to study these adaptive strategies, an accurate and convenient methodology is lacking even in model plants like *Arabidopsis thaliana*. The gradual nature of drought stress imposition protocol poses a hindrance in simultaneously applying pathogen infection under laboratory conditions to achieve combined stress. In present study we aimed to establish systematic combined stress protocol and to study physiological responses of the plants to various degrees of combined stress. Here, we have comprehensively studied the impact of combined drought and *Pseudomonas syringae* pv. tomato DC3000 infection on *A. thaliana*. Further, by employing different permutations of drought and pathogen stress intensities, an attempt was made to dissect the contribution of each individual stress effects during their concurrence. We hereby present two main aspects of combined stress viz., stress interaction and net impact of the stress on plants. Mainly, this study established a systematic protocol to assess the impact of combined drought and bacterial pathogen stress. It was observed that as a result of net impact, some physiological responses under combined stress are tailored when compared to the plants exposed to individual stresses. We also infer that plant responses under combined stress in this study are predominantly influenced by the drought stress. Our results show that pathogen multiplication was reduced by drought stress in combined stressed plants. Combined stressed plants also displayed reduced ROS generation and declined cell death which could be attributed to activation of effective basal defense responses. We hypothesize a model on ABA mediated gene regulation to partly explain the possible mechanistic basis for reduced *in planta* bacterial numbers under combined stress over individual pathogen stress.

## Introduction

Plants are continually exposed to various stress combinations in their natural habitat and amongst all, the combination of drought and pathogen infection poses a major threat to plant growth and yield (McElrone et al., 2001; Choi et al., 2013). Frequent occurrence of drought and consequently altered magnitude of pathogen infection on plants have been widely reported (Desprez-Loustau et al., 2007; Yáñez-López et al., 2012; Elad and Pertot, 2014). In spite of the wide spread impact of combined drought and pathogen stresses on plants (McElrone et al., 2001; Atkinson and Urwin, 2012; Hatmi et al., 2015; Prasch and Sonnewald, 2015; Ramegowda and Senthil-Kumar, 2015), the understanding on this interaction is primitive. Therefore, a systematic study on the impact of this combination on plants is imperative.

Combined abiotic and biotic stress results in different and convergent responses that interact and impact each other (Anderson et al., 2004; Asselbergh et al., 2008; Pandey et al., 2015). More specifically, combination of drought and bacterial pathogen stress leads to contrasting impacts on plants. For example, water deficit has been shown to cause stomata closure in the plants (Wilkinson and Davies, 2002) which may inhibit the subsequent pathogen invasion, but on the other hand some foliar bacterial pathogens open the stomata to invade the plant (Melotto et al., 2008) and this can lead to increased water loss under drought conditions. Recent studies indicate that the plants under combined stresses exhibit tailored physiological and molecular responses which are different from individual stresses (Rizhsky et al., 2004; Atkinson and Urwin, 2012; Rasmussen et al., 2013; Prasch and Sonnewald, 2015; Gupta et al., 2016). Further, existence of overlapping responses between the individual and combined stresses have also been reported (Rizhsky et al., 2004; Atkinson and Urwin, 2012; Rasmussen et al., 2013; Prasch and Sonnewald, 2015; Gupta et al., 2016). Previously, a few studies focused on exploring relationship of plant water status and foliar bacterial infection rolls out ABA as a player catering to both drought tolerance and pathogen susceptibility in plants (Mohr and Cahill, 2003). Also, ABA modulated regulation of stomata opening and closure is crucial for both pathogen defense as well as control of transpiration water loss during water deficit conditions (Lim et al., 2015). Taken together, data from wide range of studies indicate that stress interaction impact both positive and negative effects on plant responses (DiLeo et al., 2010; Ramegowda et al., 2013). However, such differential responses are yet to be explored in detail.

Plant response to combined stress depends on several factors including age (Kus et al., 2002), species or ecotypes (Prasch and Sonnewald, 2013; Rasmussen et al., 2013), and growth conditions of plants. In addition, the order of occurrence of each stressor, their intensity and duration of exposure also influence plant responses to combined stress. Severe and mild drought elicits different plant response mechanisms (Claeys et al., 2014) which lead to altered plant defenses to pathogen infection. For example, with increasing levels of drought stress, increase in susceptibility has been reported for *Vitis vinifera* inoculated with *Xylella fastidiosa* (causal agent of Pierce's disease, Choi et al., 2013). The acclimation of *Nicotiana benthamiana* to moderate drought stress reduced the *in planta* multiplication of *Pseudomonas syringae* pv. tabaci (causal agent of wildfire disease in *Nicotiana* sp.). However, severe drought stress increased the susceptibility of plants against this pathogen (Ramegowda et al., 2013). Nevertheless, influence of different drought stress levels combined with pathogen infection in plants is not well-studied. In order to understand the impact of various such interactions, a methodology to impose combined drought and pathogen stress is needed. Exploration of mechanisms imparting combined stress tolerance (both contributing to stress interaction and net impact) would be easy if studies are made in model plants which have well-annotated genetic resources and well-established pathosystem.

Here we present a method for combined imposition of drought stress and bacterial pathogen infection in *Arabidopsis thaliana*. We attempted to delineate the contribution of individual stressor in combined stress response. We found that the combined stress responses are inclined more toward the drought stress. Based on the physiological parameters assayed and few known key gene's expression profile in the study, we show that the combined stressed plants have enhanced basal defense and hence tolerate pathogen infection.

## Materials and methods

### Plant material and growth conditions

*Arabidopsis thaliana* ecotype Columbia-0 seeds (Arabidopsis Biological Resource Center, accession no. CS70000) were sown in five pots array trays (5 cm dia. pot size) at the rate of one seed per pot in a mixture of peat and vermiculite (3:1 vol/vol) and were stratified for 72 h in dark at 4°C. Plants were transferred to the environmentally controlled growth chamber (PGR15, Conviron, Canada) with diurnal cycle of 12-h-light/12-h-dark; 200 μE m^−2^s^−1^ light intensity, at 22°C temperature, 70% relative humidity and 792.9 Pa vapor pressure deficit (VPD) (calculated using http://cronklab.wikidot.com/calculation-of-vapor-pressure-deficit). Plants were bottom irrigated once in 2 days and fertilized once a week with half strength Hoaglands medium (Catalog number TS1094, Himedia, India). Potting mix preparation and plant maintenance are detailed in Video S1 and Data Sheet 1.

### Bacterial strain and its growth

*P. syringae* pv. tomato DC3000, a host pathogen of *A. thaliana* (Mysore and Ryu, 2004) was grown in 5 mL of King's B (KB) liquid medium (King et al., 1954) supplemented with rifampicin at 50 μg/mL at 28°C from single colony with a continuous shaking of 150 revolutions per minute (rpm) for 12 h.

### Application of drought stress

*A. thaliana* (32-d-old, 12 leaf stage) with an average transpiration leaf area 32 cm^2^ (per plant) grown under well-watered condition, each in 10 g of potting mix (dried at 60°C for 4 d) was subjected to progressive drought stress by withholding water. Drought stress levels were monitored by gravimetric method (Ramegowda et al., 2013). Pots along with plants were weighed twice a day (11:00 a.m. and 5:00 p.m.) and the desired level of drought stress was maintained. Control plants were maintained at 100% field capacity (FC). For stress, plants were maintained under 20, 40, 60, and 80% FC for desired duration. The potting mix used in the study attained 20% FC in 7 d (Figure S1; Video S1). Accordingly, for imposition of different drought stress levels, plants meant for 20% FC were deprived of irrigation first at the age of 32 d. For 40, 60, and 80% FC the water was withheld at the age of 34, 36, and 37 d, respectively. By this procedure on 7^th^ day, all plants reached their respective FC at the age of 38 d. Thus, it was made sure that desired drought stress levels were attained in 7 d at the same plant age. Once pots reach destined FC they were maintained at that FC by replenishing the lost amount of water, till the end of the experiment. Drought stress imposition protocol is outlined in Figure S1, Video S1, and Data Sheet 1. FC was determined using the following formula;

$$\begin{array}{l} \text{FC}\left(\%\right)=\frac{\left(\text{WW}-\text{DW}\right)}{\text{DW}}\text{ X }100 \end{array}$$

WW-wet soil weight; DW-oven dry soil weight.

### Pathogen inoculation

*P. syringae* pv. tomato DC3000 was grown in KB liquid medium supplemented with rifampicin (50 μg/mL). Overnight (12 h) grown bacterial cells with initial optical density at 600 nm (OD_600_) = 0.4, were harvested by centrifugation at 4270 g for 10 min. Pellet containing bacterial cells was washed thrice in sterile water and diluted in 10 mL of sterile water. The suspension OD_600_ was measured and was serial diluted. These dilutions (10 μL) were plated onto KB agar plates (with rifampicin). Bacteria were counted (colony forming units, CFU) and CFU/mL for each OD_600_ was assessed. The bacterial suspension was diluted to the desired concentration (1 × 10^5^, 1 × 10^4^, 5 × 10^3^, and 2 × 10^3^ CFU/mL) and was syringe (needle-less) infiltrated into the abaxial side of 38-d-old plant leaves. All the leaves were infiltrated with 5 mL of bacterial suspension per plant. The control healthy plants were infiltrated only with sterile water (mock inoculation). Outline of the pathogen treatments is provided in Figure S2.

### Combined stress imposition

Drought stress was imposed on *A. thaliana* as per the protocol detailed in previous section. One day prior to the destined FC (38-d-old plants), pathogen was infiltrated at four different concentration (viz., 10^5^, 10^4^, 5 × 10^3^, 2 × 10^3^, CFU/mL) through abaxial side of the leaves. The time of pathogen inoculation was considered as 0 h post treatment (hpt). Upon pathogen inoculation, plants were maintained at the desired FC, by replenishing the lost amount of water, till the end of the experiment. Individual drought stressed plants and individual bacterial pathogen infected plants were separately maintained. The outline for combined stress is provided in Figure S3, Video S1, and Data Sheet 1.

### Sample harvest

At the end of treatment, individual plants had leaf area in the range of 5.5 to 7.0 cm^2^ depending upon the drought stress level. For assessment of RWC and *in planta* bacterial numbers, leaf samples were collected at 24, 48, and 72 hpt, from plants aged at 39, 40, and 41 d, respectively. For parameters involving estimation of chlorophyll and reactive oxygen species and gene expression profile, leaf samples were harvested at 24 hpt. Cell death was assayed in leaf samples collected at 72 hpt. Leaves were harvested from the third layer rosettes for downstream experiments and care was taken to select leaves at similar developmental stage.

### Evaluation of stress impact on plants

#### Relative water content

The relative water content (RWC) of each leaf was measured for plants under combined stress at 24, 48, and 72 hpt. RWC was determined following the protocol described by Flower and Ludlow (1986). Briefly, after determining the fresh weight (FW), samples were immediately hydrated, by floating on de-ionized water, to full turgidity for 24 h at 22°C temperature and turgid weight (TW) was noted. Samples were then oven dried at 60°C until they reach constant weight and dry weight (DW) was measured. RWC was calculated using the formula:

$$\begin{array}{l} \text{RWC}\left(\text{\%}\right)=\left[\left(\text{FW}-\text{DW}\right)∕\left(\text{TW}-\text{DW}\right)\right]\times 100 \end{array}$$

#### *In planta* bacterial number quantification assay

Bacterial multiplication in leaves from combined stressed and pathogen treated plants was assessed at 0, 24, 48, and 72 hpt. Each infected leaf was surface sterilized with 0.01% H_2_O_2_ for 20 s, weighed and was homogenized in 500 μL of sterile water. Upon further serial dilution in sterile water, it was plated on KB agar medium supplemented with rifampicin (50 μg/mL). Bacterial number was calculated as CFU per gram fresh weight of leaf (Wang et al., 2007). Dry and fresh weight of the leaves at different FC was measured and the correction factor with leaf disc area was employed in the calculation. For all the bacterial treatments *in planta* bacterial number was assayed up to 3 days at 24 h interval. Bacterial number was calculated as per the following formula:

$$\begin{array}{l} \text{Bacterial number CFU}∕{\text{cm}}^{2}=\\ \frac{\frac{\text{Number of colonies }\times \text{ volume of homogenate}\left(\mu \text{L}\right)\;\times \text{ dilution factor}}{\text{volume plated}}}{\text{Leaf area }\left({\text{cm}}^{2}\right)} \end{array}$$

#### Estimation of chlorophyll content

Total chlorophyll content was estimated from stressed and control plant leaves as described by Hiscox and Israelstam (1979) with few modifications. Leaves were incubated for 72 h at room temperature in dark in 4.0 mL of dimethyl sulfoxide (DMSO): acetone (1:1 vol/vol) mix. Absorbance of extracts was read using Wealtec spectrophotometer (SpectroArt 200, Wealtec Corp. Meadowvale Way Sparks, NV 89431, USA) at 645 and 663 nm. To express the readings on leaf area basis, a correction factor was established and was employed in the calculation. Chlorophyll was calculated according to Arnon's (1949) equation as given below.

$$\begin{array}{l} \text{Total chlorophyll content}\left(\mu \text{g}∕\text{c}{\text{m}}^{2}\right)\\ =\frac{\left(\text{mL solvent}\right)\times \left(20.2\;\times \text{ OD }645\right)+\left(8.02\;\times \text{ OD }663\right)}{\text{Leaf dry weight}\left(\text{mg}\right)\;\times \text{ correction factor}} \end{array}$$

#### Detection of reactive oxygen species (ROS)

Generation of total ROS in Arabidopsis leaves was detected by Diaminobenzidine tetrahydrochloride (DAB) staining as per the protocol provided by Daudi and O'Brien (2012). Briefly, leaf samples were immersed in 5 mL of 10 mM Na_2_HPO_4_ DAB solution and were incubated for 12 h in dark with gentle shaking. Following staining, leaves were boiled for 2–5 min in bleaching solution (ethanol: acetic acid: glycerol = 3:1:1). Photographs were taken using a digital camera (Nikon Digital sight DS-Rs1) mounted on a Nikon Strereozoom AZ100 Microscope. ROS quantification based on image intensity was performed using ImageJ software (http://imagej.nih.gov/ij/).

#### Cell death quantification assay

Cell death assay was performed as described in Koch and Slusarenko (1990) with some modifications. Infected leaves were boiled for 2 min in trypan blue staining solution (0.02 g trypan blue, 8% phenol, 8% glycerol, 8% lactic acid, 8% water, 67% 95% ethanol). This was followed by overnight de-staining in chloral hydrate. Cell death was observed under bright field microscope. Images were captured using a digital camera (Nikon Digital sight DS-Rs1) mounted on a Nikon Strereozoom AZ100 Microscope. Image intensity was measured using ImageJ software (http://imagej.nih.gov/ij/).

#### Quantitative real-time PCR (RT-qPCR) analysis

Leaf tissue (100 mg fresh weight) from stressed and unstressed plants was sampled and frozen. Total RNA was isolated by TriZol™ reagent (Catalog number 15596018, Invitrogen, California, USA) following manufacturer's guidelines. Total RNA quality was assessed by agarose gel electrophoresis and their purity with a NanoDrop ND-1000 spectrophotometer (Thermofischer, Massachusetts, USA). The RNA samples with OD ratios at 260/280 nm in the range of 1.9–2.1, and at 260/230 nm, in the range of 2.0–2.3 were used for RT-qPCR. First strand cDNA was synthesized from 5 μg of DNase treated total RNA in a reaction volume of 50 μL using Verso™ cDNA synthesis kit (Catalog number AB1453A, Thermo Scientific, Massachusetts, USA). Gene-specific primers (Table S1) were designed using Primer 3 software (Untergrasser et al., 2012). cDNA (1 μL of five-fold-diluted) and 750 nM each of the gene specific primers were added in a final volume of 10 μL and were used for RT-qPCR using SYBR Green PCR master mix (Catalog number 600882, Agilent Technologies, California, USA) in ABI Prism 7000 sequence detection system (Applied Biosystems, Massachusetts, USA). Cycle threshold (Ct) values obtained for *AtACTIN2* (AT3G18780) gene were used to normalize data. For all the RT-qPCR experiments, two independent biological replicates were included. Fold change in transcript accumulation in stressed samples relative to non-stressed samples (100% FC or mock treated plants) was calculated using 2^−ΔΔCT^ method (Livak and Schmittgen, 2001).

### Statistical analysis

All the experiments were carried out at least twice with reproducible results. The data presented are average with the standard error of biological replicates from one experiment. The number of biological replicates varied with parameter assayed and are mentioned in the legend for each figure. Significant differences were determined by Student's *t*-test (*p* < 0.05). For the parameters involving time course study, the treatment was compared against the control at indicated time points only.

## Results and discussion

### Combined drought stress and pathogen infection protocol

To understand the interaction between drought and pathogen infection and their combined impact on *A. thaliana*, an optimum combined stress imposition protocol is warranted. Coinciding the drought stress and pathogen infection at the same time using currently available protocols is challenging. One of the main reasons for this difficulty is because the water lost by plant through transpiration leading to drought realization is gradual, however, pathogen infiltration and corresponding disease development is comparatively faster. Most prevalent methods of drought stress imposition involve dry down method to bring plants at specific field capacity (FC). Generally to understand influence of drought stress on pathogen infection, plants are allowed to reach different FC at different times and maintained continuously at respective FC's until the last set reach severe stress (for example, 20% FC) (Ramegowda et al., 2013). In this protocol the drought stress level and duration varies and could not be directly applied to *A. thaliana*. In order to keep uniform stress levels, in this study, number of days required to reach each FC was standardized initially. Accordingly, water withholding for the severe stress level was initiated first and then later for less severe stresses (Figure S1). We thus achieved the drought stress levels without altering the stress duration and synchronized to plant age. Moreover, by using the potting mixture that can lose water quickly and by exploiting the environmental variables namely, relative humidity, vapor pressure deficit (VPD) and air flow in growth chamber, we were able to minimize the time period required for the pots from 100% FC to reach desired drought stress. This paved way for combining drought stress imposition and pathogen infection in plants. Based on this, we superimposed drought and pathogen stress onto plants and developed a novel protocol for combined stress imposition (Figure S3). Briefly, in this protocol, drought stress was imposed by gravimetric method and its realization by plant was assessed by measuring the relative water content (RWC). We observed that with an increase in the intensity of drought stress viz., at soil water status 80, 60, 40, and 20% FC, plants exhibited reduction in RWC of 78, 65, 55, and 35%, respectively in comparison to the RWC of 92% in well-watered control plants (Figure S4A). We maintained plants at the same drought level for another 3 days for combined stress study. Low turgor in the leaf resulted in wilting symptoms in drought stressed plants (Figure S4C). The wilting symptoms were prominent in the plants under severe stress i.e., at FC 40% and FC 20% (Figure S4C).

**Figure 1:**
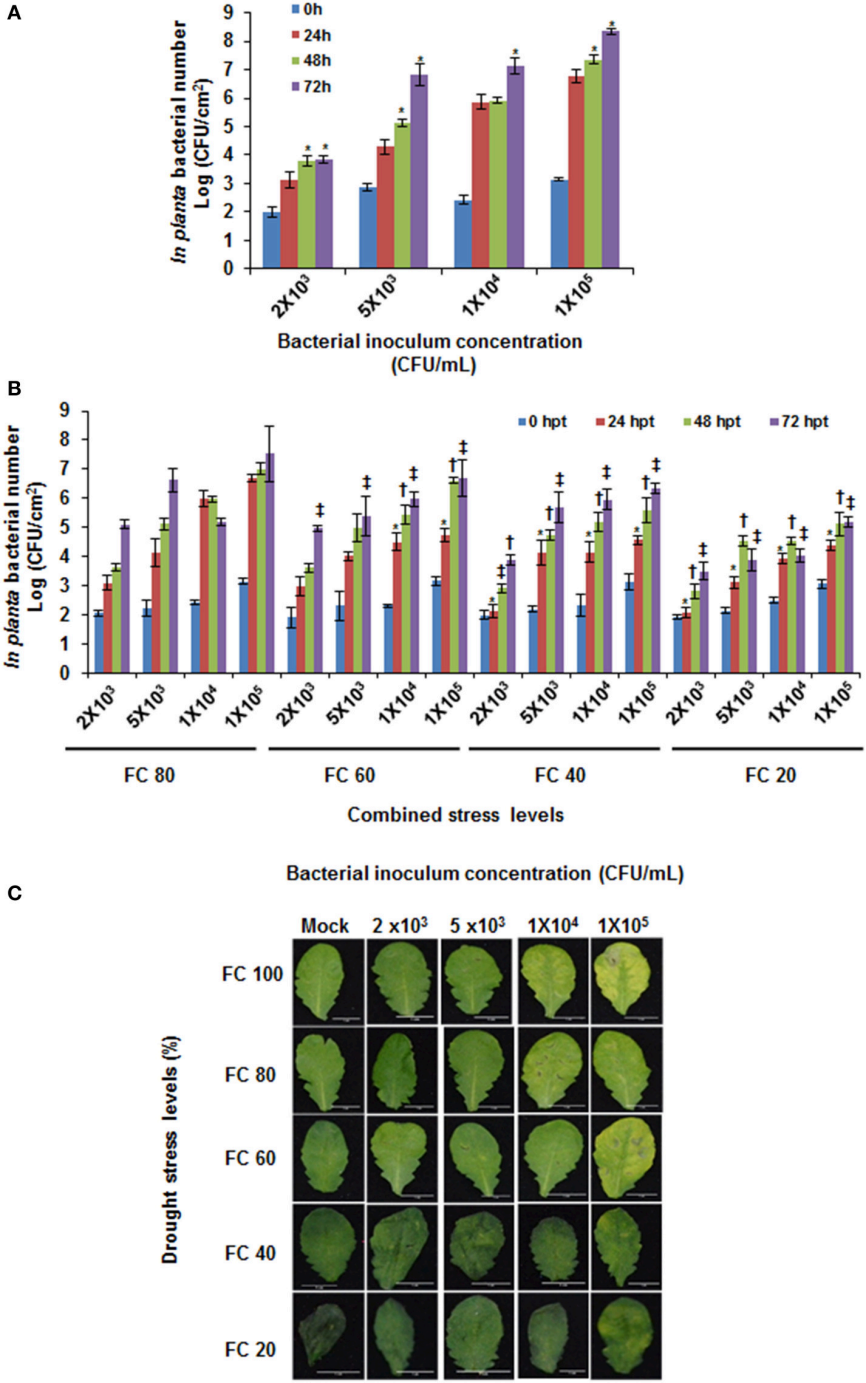
*****In planta*** bacterial multiplication in individual and combined stressed plants**. Different concentrations of *Pseudomonas syringae* pv. tomato DC3000 were syringe inoculated on to *A. thaliana* maintained under well-watered **(A)** and at different drought stress levels (**B**). The *in planta* bacterial number was recorded at 0, 24, 48, and 72 hpt. 0 hpt values indicate initial pathogen inoculum infiltrated at the start of the combined stress experiment **(A,B)**. Phenotypic symptoms (for example, chlorosis) in leaves from these plants were recorded at 72 hpt **(C)**. Water only infiltrated plants (mock treatment) were maintained for comparison with combined drought and pathogen stressed plants. Data represents the mean of three biological replicates (*n* = 3) and error bars show ± standard error of mean (SEM). Statistical significance using Student's *t* test was calculated over different time periods against bacterial multiplication at 24 hpt. Symbol * in **(A)** denotes significance at *p* < 0.05. Symbols *, †, and ‡ in **(B)** denotes significance for 24, 48, and 72 hpt, respectively over respective pathogen concentration (FC100%) at each time point at *p* < 0.05. X-axis in **(B)** represents bacterial concentration as CFU/mL and field capacity (%) together as combined stress. The experiment was repeated thrice and consistent results were observed. FC, field capacity (%); hpt, hours post combined stress treatment.

In the combined stress protocol, 1 day before plants were to reach designated drought levels, they were inoculated with different concentrations of pathogen (Figure S3). Such that, at 24 h post combined stress treatment (hpt) plants would experience combined drought and pathogen stress. In this study, 24 hpt was considered as the minimal time point for plants to adopt physiological changes in response to combined stress and in this purview physiological parameters were assayed at this time point. It is perceived that drought stress is known to reduce stomata opening (Cowan and Farquhar, 1977; Chaves et al., 2003). Since this could cause variation in the initial pathogen inoculum in severe stressed plants, we used syringe inoculation wherein the pathogen is directly placed in apoplast. Further, the initial *in planta* bacterial numbers infiltrated into the leaves from plants at different drought levels were found to be same as shown at 0 h post pathogen inoculation (Figure 1).

The two stressors while interacting with each other can provoke novel plant response that is not observed under individual stress or control plants (Rizhsky et al., 2004; Atkinson and Urwin, 2012; Rasmussen et al., 2013; Prasch and Sonnewald, 2015; Gupta et al., 2016). Previously Ramegowda et al. (2013) studied the influence of drought stress on pathogen infection, wherein the factors responsible for imparting tolerance against pathogen were quantified only in drought stressed plants. With a view to delineate specific plant responses toward net effect of combined stress, several intensities of combined stress covering various drought and pathogen stress levels were tested in the current study. We combined different drought levels and different time points (& similarly for pathogen) and this at a certain state ensured concurrence of two stresses. We referred this as combined stress as explained previously in the literature (Ramegowda and Senthil-Kumar, 2015). Results from RWC measurements and *in planta* bacterial number assay indicated successful drought stress imposition and pathogen infection, respectively, in combined stressed plants (Figure S4B, Figure 1B). The combined stressed plants with mild drought stress (FC 80%) level showed more disease symptoms in comparison to the plants under severe drought stress (FC 20%) when infiltrated with similar pathogen inoculum (Figure 1C). At higher drought levels, no disease symptoms were observed (at 20% over 80% FC; Figure S4). This indicates plant responses under combined stress depends on severity of stresses. Thus, in order to study one particular parameter, an optimum combined stress with mild drought level at FC 60% and pathogen inoculums of 1 X 10^4^ CFU/mL should be considered.

### Reduced pathogen number in plants under combined stress

The interaction between two stressors can alter the plant responses toward one particular stress. Bacterial infection process consists of two phases, one is marked by external environment on leaf surface. The bacteria thrive in large number on leaf surface and are exposed to water, temperature and solar radiations stresses. These factors influence their survival on leaf surface. Second, upon entry into the leaves, bacteria thrives in the apoplast and experience severe water limitation. During this phase, bacteria up-regulates the genes involved in synthesis and regulation of alginates and compatible solutes like trehalose. This enables bacteria to sustain the level of drought stress in apoplastic spaces (Yu et al., 2013). Additionally, the plant water status influences *in vivo* multiplication of pathogen (Wright and Beattie, 2004; Freeman and Beattie, 2009). *P. syringae* is a water loving bacteria and multiplies under high moisture conditions. Upon infection, it senses the plant water level and causes water soaked lesions (Peterson, 2009). However, upon pathogen infection leaf water status plays a key role in determining the outcome of pathogen infection. At the time of infection, plants respond to *P. syringae* by complete inhibition of vascular water movement into the infection sites and increasing the leaf water transpiration, in this way plant restricts multiplication of avirulent bacteria by starving it for water (Freeman and Beattie, 2009). These scenarios are expected to completely change in a drought stressed plant. In order to dissect such responses, we studied the pathogen multiplication in infected plants under varied drought stress levels in comparison with the pathogen inoculated plants. Control plants infiltrated with different pathogen concentrations showed increased *in planta* bacterial number at all three time points tested, reflective of a dose dependent compatible host-pathogen interaction (Figure 1A). In plants under combined stress, pathogen multiplication was reduced as severity of drought stress increased (Figure 1B). Plants inoculated with pathogen at 1 × 10^5^ CFU/mL showed 0.6 fold reduction in the *in planta* bacterial number under severe drought stress (FC 20%) as compared to the plants maintained at mild drought stress (FC 80%; Figure 1B). Also, the extent of chlorotic area in leaves was exacerbated with the increased *in planta* multiplication of bacteria in pathogen inoculated plants (Figure 1C, Figure S5). However, the chlorotic symptoms sublimed in the plants at severe drought stress in comparison to the plants at mild drought stress at 72 hpt (Figure 1C, Figure S5). Comparison of this data with the RWC in combined stressed plants indicated that relatively less water in apoplastic spaces in plants under drought stress possibly have induced basal defense against oncoming pathogen stress. In addition, this scenario might have deprived water availability to the pathogen (Beattie, 2011).

### Differential impact of combined stress and individual stress on *A. thaliana*

**Figure 2:**
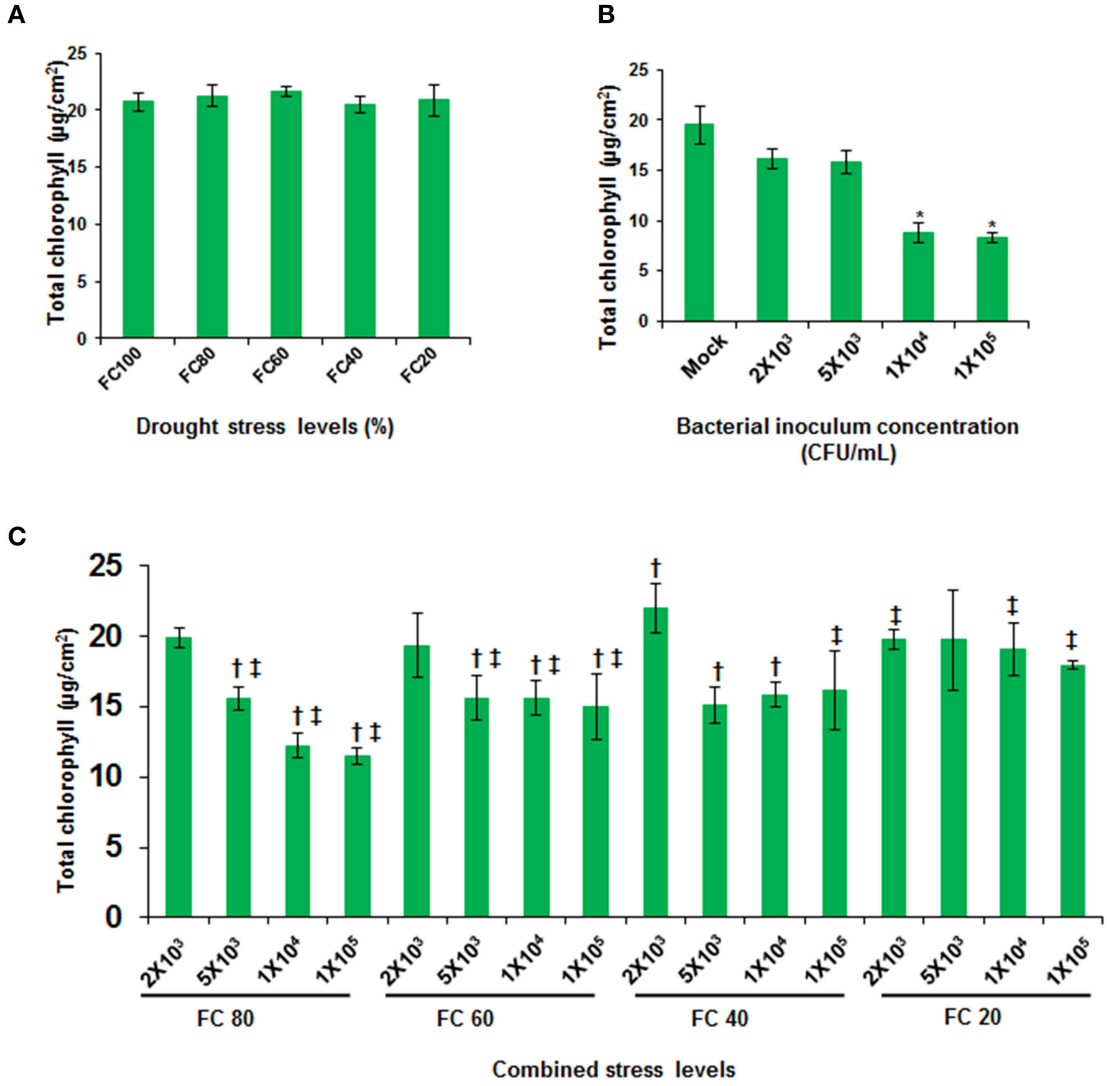
**Total chlorophyll content in individual and combined stressed plants**. Total chlorophyll content in *A. thaliana* leaves was assayed across different drought stress levels **(A)**, in leaves inoculated with different pathogen concentrations **(B)** and leaves from combined stressed plants **(C)**. All data points were obtained at 24 hpt. They represent the mean of six biological replicates (*n* = 6) and error bars show ± SEM. Statistical significance was calculated using Student's *t* test. * represents significant difference in pathogen inoculated plants over mock treated, and symbols † and ‡ denotes significance for combined stressed plants over individual drought stressed and pathogen infected plants, respectively at *p* < 0.05. Data from drought stressed plants should be compared with control plant at 100% FC and pathogen inoculated plants should be compared with mock infiltrated plants. The experiment was repeated twice and similar results were seen. X-axis in **(C)** represents bacterial concentration as CFU/mL and field capacity (%) together as combined stress. FC, field capacity (%); hpt, hours post combined stress treatment.

Few focused studies on concurrent stresses in plants revealed that plants provoke altogether different plant responses which were not seen earlier under either of the individual stress (Xu et al., 2008; Atkinson et al., 2013; Prasch and Sonnewald, 2013; Gupta et al., 2016). As a result of interaction among drought, pathogen and plant, a new net effect of combined stress could be anticipated. In order to study the net effect of combined stress, the impact of stress on plants under combined stress was compared with the individual- and non-stressed plants by estimating total chlorophyll and cell death. Plants at mild and moderate drought stress levels did not show statistically significant change in total chlorophyll content as compared to well-watered plants (Figure 2A). In agreement with these observations, previous transcriptome studies showed a non-significant effect of mild drought on the expression of photosynthesis related genes (Chaves et al., 2009) and this indicates that photosynthesis is not severely affected by mild and moderate drought stress (Cornic and Massacci, 1996; Flexas and Medrano, 2002). However, the pathogen infected plants showed up to 42% reduction in chlorophyll over mock inoculated plants during disease progression (Figure 2B). The reduction was indicative of the pathogen-induced disease (Figure 1C; Katagiri et al., 2002). Combined stressed plants showed tailored response in terms of total chlorophyll content when compared to individual stressed plants (Figure 2C). Plants infiltrated with pathogen (1 × 10^5^ CFU/mL) at 60% FC, showed 31% reduction in total chlorophyll over drought stressed plants (FC 60% only plants) but, showed better retention of chlorophyll content over pathogen (1 × 10^5^ CFU/mL) inoculated plants (Figure 2C). Overall the decrease in chlorophyll content was influenced by pathogen concentration more at mild drought levels (Figure 2C).

**Figure 3:**
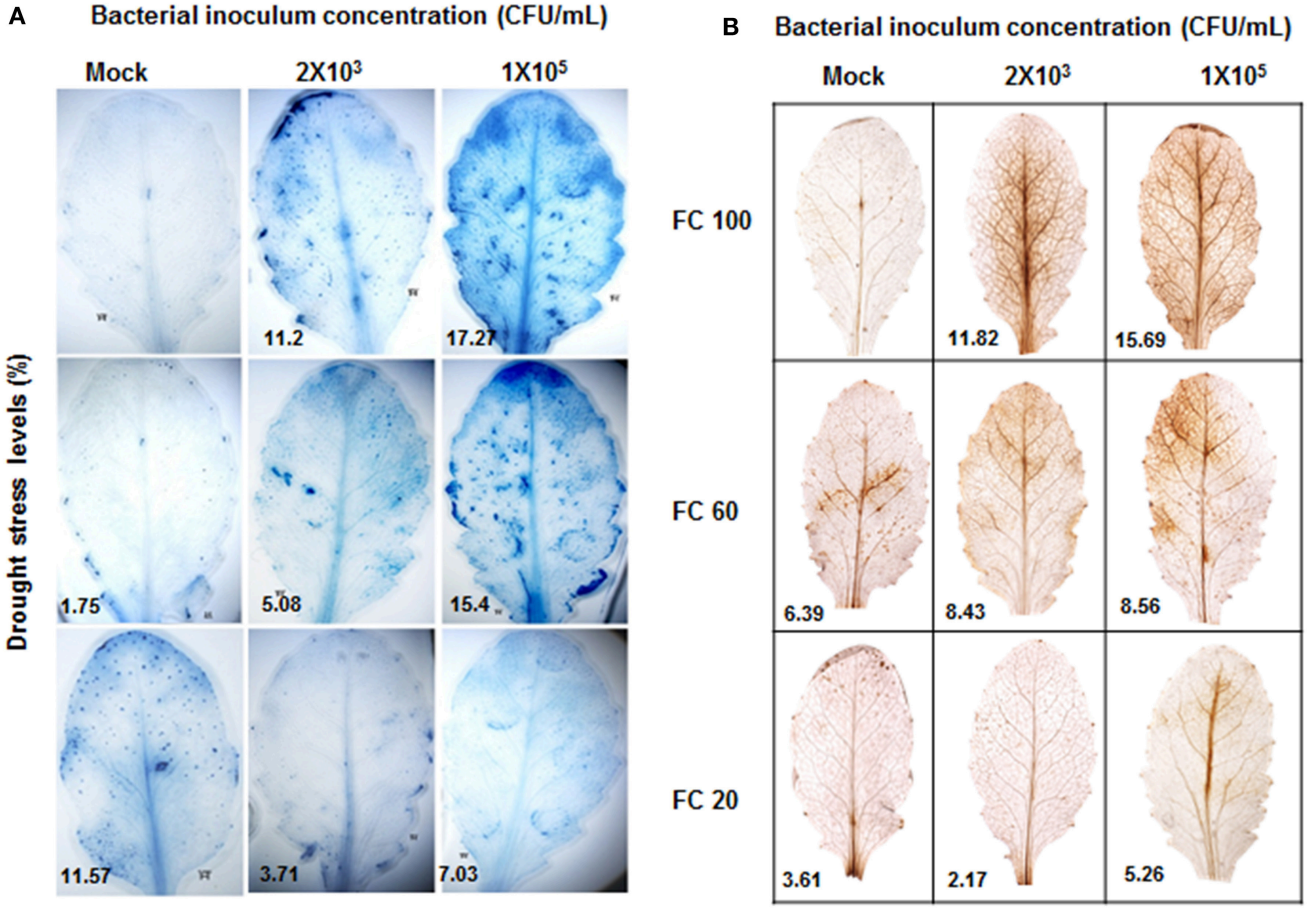
**Extent of cell death and ROS generation in individual and combined stressed plants**. Cell death was estimated at 72 hpt by trypan blue staining and photographs were taken **(A)**. Stained area was scored by ImageJ software (http://imagej.nih.gov/ij/) and was expressed as fold change over control. DAB stained leaves showing ROS are presented **(B)**. Each experiment was carried out with three biological replicates.

*P. syringae* pv. tomato DC3000 infection impairs plant machinery and lead to necrotrophic cell death (Katagiri et al., 2002). Severe drought stress also results in cell death due to excessive ROS generation and other factors that impair metabolic activity. Drought stressed plants showed cell death at 20% FC (Figure 3A). Pathogen infected leaves showed cell death in concentration dependent manner (Figure 3A). Combined stressed plants exhibited reduced cell death in comparison to the pathogen inoculated plants (Figure 3A). Our results also demonstrated that the pattern of ROS generation was in accordance with the noted trend in cell death (Figure 3B). Overall, the reduction in chlorophyll content and extent of cell death in combined stressed plants were less compared to individual stressed plants showing increased resistance of plants under combined stress. These results implicate the unique nature of combined stress effect and responses as compared to individual stresses (Table S2).

### Induction of drought and pathogen defense related genes under combined stress

**Figure 4:**
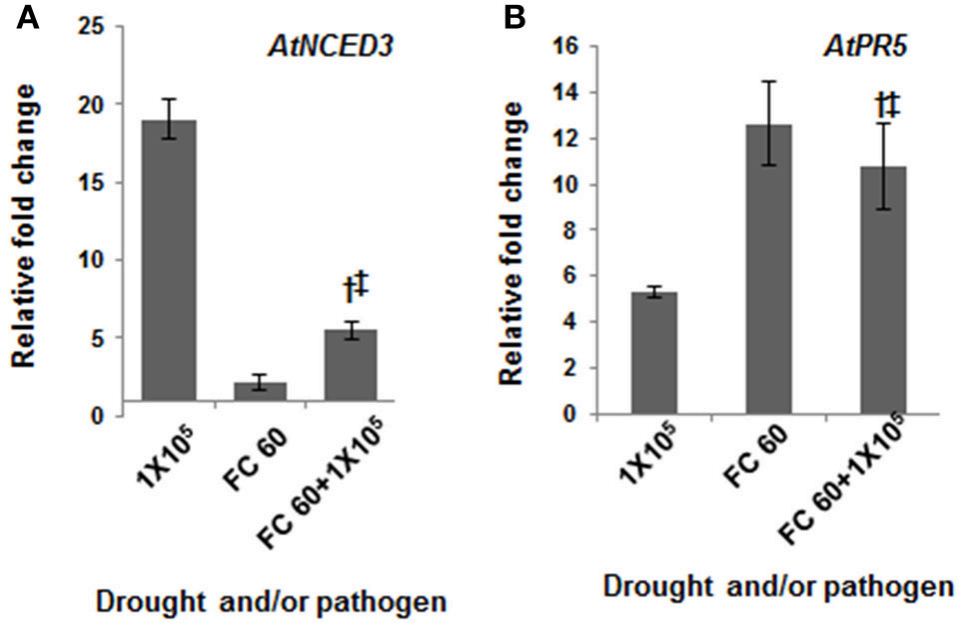
**Transcript expression of drought and pathogen stress responsive genes in individual and combined stressed plants**. Transcript expression was analyzed by RT-qPCR in drought stressed (FC 60%), pathogen infected (10^4^ CFU/mL) and combined stressed (FC 60% + 10^4^ CFU/mL) plants. Relative transcript expression levels of **(A)** *AtNCED3* and **(B)** *AtPR5* was assessed at 24 hpt. Data represents the mean of two biological replicates (*n* = 2) and error bars show ± standard error of mean (SEM). Statistical significance using Student's *t* test was calculated over individual stresses and symbols † and ‡ denotes significance for combined stressed plants over individual drought stressed and pathogen inoculated plants, respectively at *p* < 0.05. The experiment was repeated twice with similar results. FC, field capacity (%); hpt, hours post combined stress treatment.

In order to identify the optimum level of drought and pathogen stressors during combined stress and to understand the possible molecular mechanism during stress interaction, expression pattern of a few known genes involved in individual drought and pathogen stress responses were studied. Under drought stress, pathogen infection and combined stress, the expression profile of drought stress responsive genes encoding late embryogenesis abundant protein (*AtLEA4*), 9-cis-epoxycarotenoid dioxygenase (*AtNCED3*), dehydration responsive element binding factor (*AtDREB1A*), and pathogen stress responsive gene encoding pathogenesis related protein (*AtPR1* and *AtPR5*), phenylalanine ammonia lyase (*AtPAL1*) and non-expresser of PR genes 1 (*AtNPR1*) were studied. *AtLEA4*, a gene encoding a chaperon protein which prevents water stress induced aggregation of sensitive proteins (Chakrabortee et al., 2007; Olvera-Carrillo et al., 2010), and *AtNCED3*, an abscisic acid (ABA) biosynthesis gene have been shown to be up-regulated during drought stress leading to accumulation of ABA (Iuchi et al., 2001). Besides, we also studied the expression pattern of leaf wilting 2 (*AtLEW2*) gene which encodes a cellulose synthase gene and is involved in secondary cell wall synthesis (Taylor-Teeples et al., 2014). This gene has previously been shown to exhibit up-regulated expression under both drought (Chen et al., 2005) and pathogen stress (Hernández-Blanco et al., 2007). The RT-qPCR analysis in current study depicted stress level dependent expression of drought stress responsive genes in drought stressed plants with the highest expression in severe drought stressed plants (Figure 4, Figure S6). Plants maintained at severe drought stress of 20% FC showed ~24, 19, and 13-fold induction in *AtLEA4, AtNCED3* and *AtDREB1A* gene transcript expression, respectively over well-watered control plants. However, plants that experienced mild drought level of FC 80% exhibited only 1.6, 1.7, and 1.5-fold changes in *AtLEA4, AtNCED3*, and *AtDREB1A* gene expression, respectively (Figure S6A). Likewise the expression profile of defense genes exhibited a pathogen inoculum dependent trend. Plants inoculated with 1 × 10^5^ CFU/mL showed 74, 10, and 15-fold inductions in *AtPR1, AtPR5*, and *AtPAL1* genes, respectively. Plants inoculated with 2 × 10^3^ CFU/mL showed an unaltered expression of *AtPR1, AtPAL1*, and 1.8-fold change of *AtPR5* gene (Figure S6B). Similar trend was observed under the combined stress, wherein the expression of all these genes increased with increase in severity of combined stress (Figure S6C). Thus, our transcript expression data indicated that plants indeed experienced the drought stress and pathogen infection. We also noted the induction of pathogen defense responsive genes under drought stress and vice-versa. This is in line with the previous reports (Zhu et al., 1995; Seo et al., 2008; Liu et al., 2013; Ramegowda et al., 2013). We observed that *AtPR5* exhibited greater fold induction in drought and combined stressed plants over pathogen stressed plants. It is worth noting here that *AtPR5* encodes osmotin protein which provides tolerance under drought stress (Zhu et al., 1995; Seo et al., 2008). Similarly, at severe stress intensities, the induction of *AtNCED3* gene was high in pathogen or combined stressed plants compared to drought stressed plants. Reportedly, extract from drought stressed leaves, through accumulation of PR proteins and defense response elicitors was able to control the *Diplocarpon rosae* infection in rose (Gachomo and Kotchoni, 2008). *P. syringae* exploit effector mediated induction of *AtNCED3* which results in enhanced ABA accumulation and bacterial colonization (de Torres-Zabala et al., 2007). During combined stress, the *AtNCED3* gene induction might lead to accumulation of ABA, which in turn represses *AtNAC* gene transcript expression. Gene product of AtNAC1, a transcription factor has been reported to repress *PR* gene expression (Delessert et al., 2005). *AtNAC1* gene expression was up regulated under drought stress (Tran et al., 2004) but down-regulation under combined stress. The noted down-regulation of *AtNAC1* and activated expression of *AtPR1* or *AtPR*5 during combined stress in support of literature information indicates the loss of *AtNAC1* mediated suppression of *AtPR5* gene expression. In order to strengthen the proposed mechanism, we examined the response of *atnac6* mutant toward combined stress. AtNAC6 is another member from family of NAC transcription factors and could modulate ABA mediated plant response toward stress (Balazadeh et al., 2010; Yang et al., 2011). In our earlier study involving microarray based transcriptome analysis in *A. thaliana* under combined drought and pathogen stress, we found *AtNAC6* was up-regulated (Gupta et al., 2016). Our preliminary results revealed higher *in planta* bacterial number in *atnac6* mutant plants under combined stress compared to mutant plants infected with only pathogen (Figure S7). Further, the fold reduction in bacterial number in combined stressed wild-type plants compared to pathogen only stressed plants was not seen in the mutant plants. Altogether, our results thus hint that plants adopt a tailored strategy during combined stress. Accordingly, under combined stress, activation of basal plant defenses involving *AtPR5* and *AtNCED3* gene induction might have contributed to the reduced bacterial multiplication. Moreover, the increased tolerance of *A. thaliana* under combined drought and bacterial stress can be attributed to a balance maintained between ABA and salicylic acid mediated signaling pathways. From this, we propose that the tailored responses under combined stress cannot be extrapolated from individual stress experiments.

When plant basal defense responses are already active under drought stress, in order for the pathogen to establish itself, it suppresses the basal defense responses by releasing effectors into the plant cell. For example, *P. syringae* type III effector HopAM1 enhances the virulence (of an avirulent pathogen) on water stressed plants (Goel et al., 2008). Existing literature on studying plant-bacterial interaction in presence of drought stress (Table S3) indicates that in response to drought stress, not only bacterial multiplication is high but the plant growth and biomass are reduced. In this regard drought stress induced endogenous ABA levels has been proposed as a factor driving plant more susceptible to the pathogen (Mohr and Cahill, 2003). However, in the present study we observed reduced bacterial number in combined drought and pathogen stressed plants which could be attributed to differential regulation of downstream processes.

## Conclusions

The combination of drought and bacterial pathogen infection is an agronomically important and altogether new stress. We studied various stress intensities of drought and pathogen combination by overlaying the drought stress with pathogen infection. Based on the physiological markers and the expression of endogenous genes that respond to changes in plant water status and pathogen infection, we established the much needed optimum combined stress protocol. We have tried to dissect the combined stress influence on the plants first to understand influence of one stressor over other in the plant interface. Second the net impact of combined stress on plants was revealed. The first part, i.e., effect of drought in increasing/ decreasing *in planta* bacterial multiplication could be explained in terms of requirement of water for *P. syringae* infection. Furthermore, interaction between two stressors can modulate final response of the plant resulting into net impact, which may lead to susceptibility or a response similar to control plants. So, from this perspective it is useful to study combined drought stress and *P. syringae* interaction in *A. thaliana*. The study also reflects that choice of combined stress level should be based on parameter to be studied. For example, we found that in order to study the net impact of pathogen interaction with drought in combined stress plants, moderate combined stress levels should be used. From this study we infer that drought stress provides endurance toward the oncoming pathogen infection in plants which could be attributed to ABA altered defense pathways (Figure S8). In comparison to individual stressed plants, combined stressed samples exhibited greater fold induction in drought and pathogen specific marker genes and thereby revealing a tailored response.

## Author contributions

MS conceived the idea. MS and AG designed the study. AG executed the experiments, analyzed the data. SD contributed to plant handling, chlorophyll estimation and phenotypic observations. MS and AG wrote the manuscript.

### Conflict of interest statement

The authors declare that the research was conducted in the absence of any commercial or financial relationships that could be construed as a potential conflict of interest.

## Supplementary material

Video S1 **Combined stress imposition protocol in *A. thaliana***. We developed a shorter drought imposition protocol and coincided drought stress and *P. syringae* pv. tomato DC3000 infection in *A. thaliana* to realize combined stress. One day before the plants were to achieve their respective FCs, the abaxial surface of the leaves (drought stressed) was syringe infiltrated with *P. syringae* DC3000. The combined stress impact was scored for 3 days after stress imposition.
