# Supplementary material for: Drought Stress Predominantly Endures Arabidopsis thaliana to Pseudomonas syringae Infection
Source: Front Plant Sci. 2016 Jun 7;7:808. doi: 10.3389/fpls.2016.00808 (PMC4894909; doi:10.3389/fpls.2016.00808)
Supplement: Supplementary file 2 [file Table1.DOC]

| **Gene ID** | **Oligo name** | **Oligo Seq 5’ – 3’** |
| --- | --- | --- |
| AT1G75040 | *AtPR5*-F | ATCTCCAGTATTCACATTCTC |
|  | *AtPR5*-R | ACCTGGAGTCAATTCAAATC |
| AT2G14610 | *AtPR1*- F | TTGTAGGTGCTCTTGTTCTTC |
|  | *AtPR1*- R | CTCTTAGTTGTTCTGCGTAGC |
| AT3G14440 | *AtNCED3*-F | CAGCCGCCATTATCGTCTTC |
|  | *AtNCED3*-R | TAACAACAATGGCGGGAGAG |
| AT2G37040 | *AtPAL1*- F | GCAGCGGAGCAAATGAAAGG |
|  | *AtPAL1*- R | ACCAATAGTTGAGATCGCAGCCAC |
| AT4G25480 | *AtDREB1A*- F | GGCTCCGATTACGAGTCTTCG |
|  | *AtDREB1A*- R | CCCACTTACCGGAGTTTCTCC |
| AT4G18780 | *AtLEW2*-F | ATGATGGAGTCTAGGTCTCCCATC |
|  | *AtLEW2*-R | CGCAACGCAAGCAAATTCTTC |
| AT1G32560 | *AtLEA4*-F | ATGGCTAGTACAGCCAAGGAG |
|  | *AtLEA4*-F | GTGCATATCCATATTCGCTTCT |
| AT1G64280 | *AtNPR1*-F | GAATCCGTCTTTGACTCGCC |
|  | *AtNPR1*-R | GCGGTGTTGTTGGAGTCTTT |
| AT3G15170 | *AtNAC1*-F | TGGCGTTTGGTCAGTTTCTG |
|  | *AtNAC1*-R | AGAGAGTAAACGGCCACACA |
| AT3G62250 | *AtUBQ5*- F | ATCCGACACCATCGACAATG |
|  | *AtUBQ5*- R | TGTTGTAGTCGGCGAGGGTAC |
| AT3G18780 | *AtACTIN2*-F | ATTCTTGCTTCCCTCAGCAC |
|  | *AtACTIN2*-R | CCCCAGCTTTTTAAGCCTTT |

**Table S1.** List of primers used in the study.
