# Supplementary material for: Drought Stress Predominantly Endures Arabidopsis thaliana to Pseudomonas syringae Infection
Source: Front Plant Sci. 2016 Jun 7;7:808. doi: 10.3389/fpls.2016.00808 (PMC4894909; doi:10.3389/fpls.2016.00808)
Supplement: Supplementary file 3 [file Table2.DOC]

**Table S2.** Visual inspection score for phenotypic effect caused by drought, *Pseudomonas syringae* pv. tomato DC3000 and their combination on *A. thaliana*

| **Stress treatment (72 hpt)** | **Plant growth (leaf area)** | **Chlorosis** | **Wilting** |
| --- | --- | --- | --- |
| Non stressed plant | | | |
| FC100٪ | +++++ | - | - |
| Drought stress | | | |
| FC20٪ | + | - | ***** |
| FC40٪ | ++ | - | **** |
| FC60٪ | +++ | - | *** |
| FC80٪ | ++++ | - | * |
| Pathogen stress | | | |
| FC100٪ (M) | +++++ | - | - |
| FC100٪+10^6^ cfu/ml | ++++ | ***** | - |
| FC100٪+10^5^cfu/ml | +++++ | *** | - |
| FC100٪+5X10^4^cfu/ml | +++++ | * | - |
| FC100٪+2X10^4^cfu/ml | +++++ | - | - |
| Combined stress | | | |
| Drought + pathogen |  |  |  |
| FC20٪ (M) | + | - | ***** |
| FC40٪ (M) | ++ | - | **** |
| FC60٪ (M) | +++ | - | *** |
| FC80٪ (M) | ++++ | - | * |
| FC20٪+10^6^cfu/ml | + | ** | ***** |
| FC20٪+10^5^cfu/ml | + | * | ***** |
| FC20٪+5X10^4^cfu/ml | + | - | ***** |
| FC20٪+2X10^4^cfu/ml | + | - | **** |
| FC40٪+10^6^cfu/ml | ++ | ** | **** |
| FC40٪+10^5^cfu/ml | ++ | ** | **** |
| FC40٪+5X10^4^cfu/ml | ++ | - | **** |
| FC40٪+2X10^4^cfu/ml | ++ | - | **** |
| FC60٪+10^6^cfu/ml | +++ | *** | *** |
| FC60٪+10^5^cfu/ml | +++ | ** | *** |
| FC60٪+5X10^4^cfu/ml | +++ | + | *** |
| FC60٪+2X10^4^cfu/ml | +++ | - | *** |
| FC80٪+10^6^cfu/ml | +++ | **** | * |
| FC80٪+10^5^cfu/ml | ++++ | ** | * |
| FC80٪+5X10^4^cfu/ml | ++++ | * | * |
| FC80٪+2X10^4^cfu/ml | ++++ | - | * |

- no chlorosis, wilting (visual inspection observed over control)

M, mock; FC, field capacity; cfu- colony forming unit

Plant area at (measured through ImageJ software) +++++ =~ 36cm^2^ ,++++= ~31 cm^2^ ,+++= ~ 24 cm^2^,++= ~18cm^2^,+=~16 cm^2^

*****>****>***>**=order of chlorosis/wilting maximum to minimum
