# Supplementary material for: Drought Stress Predominantly Endures Arabidopsis thaliana to Pseudomonas syringae Infection
Source: Front Plant Sci. 2016 Jun 7;7:808. doi: 10.3389/fpls.2016.00808 (PMC4894909; doi:10.3389/fpls.2016.00808)
Supplement: Supplementary file 4 [file Table3.DOCX]

**Table S3** Evidences for variations in plant-pathogen interaction under drought stress.

| Plant species | First stress | Second stress | Plant response | Reference |
| --- | --- | --- | --- | --- |
| *Parthenocissus quinquefolia* | Drought stress | *Xylella* *fastidiosa* | Enhanced severity and progression of disease symptoms | McElrone et al., 2003 |
| *Nicotiana benthamiana* | Drought stress (60% FC) | *P. syringae* pv. tabaci | Reduced bacterial multiplication | Ramegowda et al., 2013 |
| *Nicotiana benthamiana* | Drought stress (60% FC) | *P. syringae* pv. tabaci | Reduced disease symptoms and reduced bacterial multiplication | Ramegowda et al., 2013 |
| *Nicotiana benthamiana* | Drought stress (20% FC) | *P. syringae* pv. tabaci | No change | Ramegowda et al., 2013 |
| *A. thaliana* | Drought stress | *P. syringae* pv tomato (avirulent isolate) | Increased susceptibility of plants | Mohr and  Cahill, 2003 |
| *A. thaliana* | Drought stress (mild) | *P. syringae* pv tomato | Increased susceptibility of plants | Fan et al., 2009 |
| *Vitis vinifera* | Drought stress | *Xylella* *fastidiosa* | Severe disease symptoms | Choi et al., 2013 |

References

McElrone, A.J., Sherald, J.L., Forseth, I.N. (2003). Interactive effects of water stress and xylem-limited bacterial infection on the water relations of a host vine. *J. Exp. Bot.* 54, 419-430. doi: 10.1093/jxb/erg046

Ramegowda, V., Senthil-Kumar, M., Ishiga, Y., Kaundal, A., Udayakumar, M., Mysore, K.S. (2013). Drought stress acclimation imparts tolerance to *Sclerotinia sclerotiorum* and *Pseudomonas syringae* in *Nicotiana benthamiana*. *Int. J. Mol. Sci*. 14, 9497–9513. doi: 10.3390/ijms14059497

Mohr, P.G., Cahill, D.M. (2003). Abscisic acid influences the susceptibility of *Arabidopsis* *thaliana* to *Pseudomonas syringae* pv. tomato and *Peronospora parasitica*. *Funct. Plant Biol*. 30, 461–469. doi:10.1071/FP02231

Choi, H-K., Iandolino, A., Goes da Silva, F., Cook, D. (2013). Water deficit modulates the response of *Vitis* *vinifera* to the Pierce’s disease pathogen *Xylella fastidiosa*. *Mol*. *Plant Microbe Interact*. 26, 643–657. doi: 10.1094/MPMI-09-12-0217-R

Fan, J., Hill, L., Crooks, C., Doerner, P., Lamb, C. (2009). Abscisic acid has a key role in modulating diverse plant-pathogen interactions. *Plant Physiol*. 150, 1750-1761.
