# Supplementary material for: Drought Stress Predominantly Endures Arabidopsis thaliana to Pseudomonas syringae Infection
Source: Front Plant Sci. 2016 Jun 7;7:808. doi: 10.3389/fpls.2016.00808 (PMC4894909; doi:10.3389/fpls.2016.00808)
Supplement: Supplementary file 5 [file Presentation1.PPTX]

## Slide 1
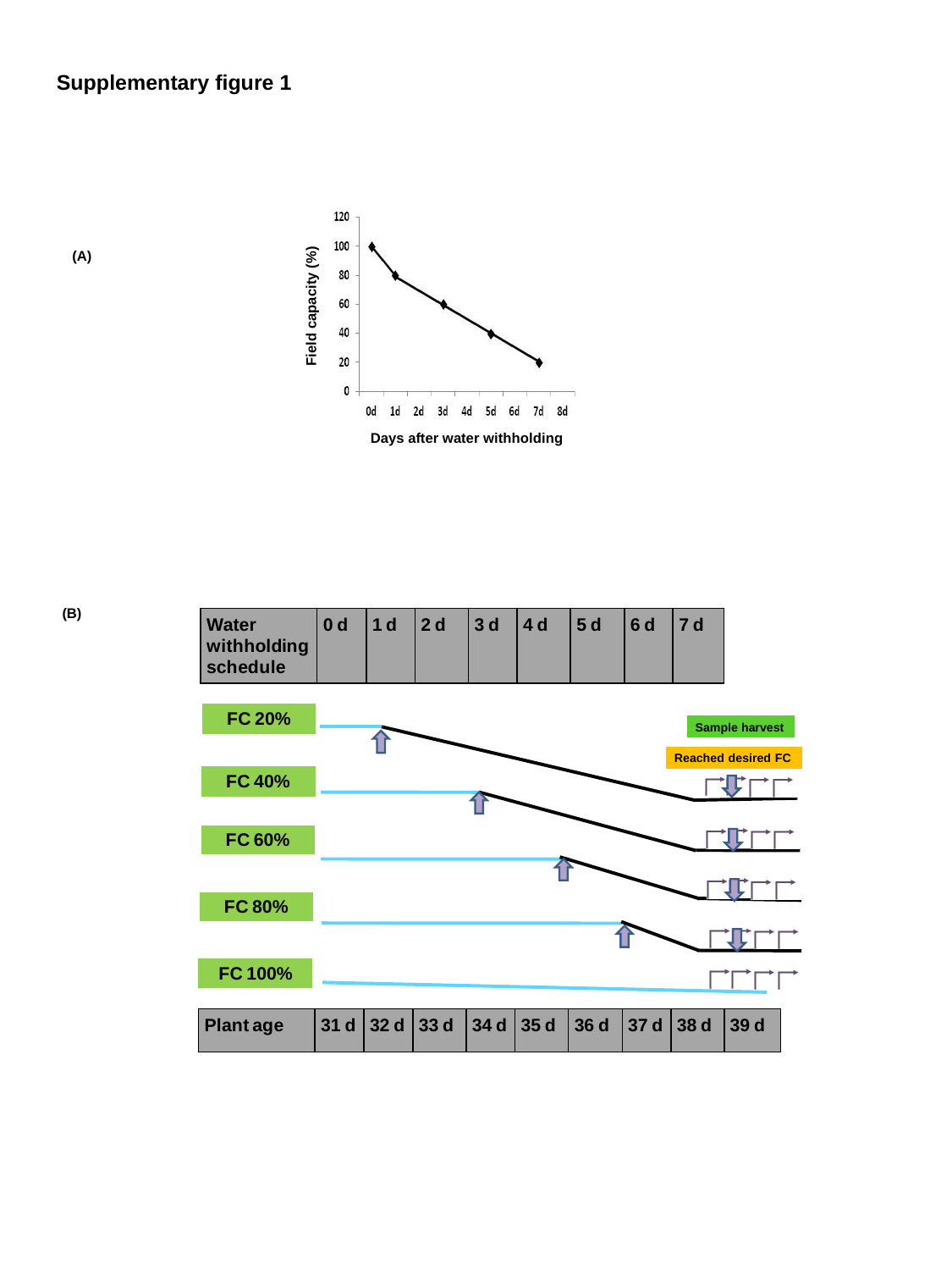

Supplementary figure 1
Field capacity (%)
Days after water withholding
(B)
(A)

## Slide 2
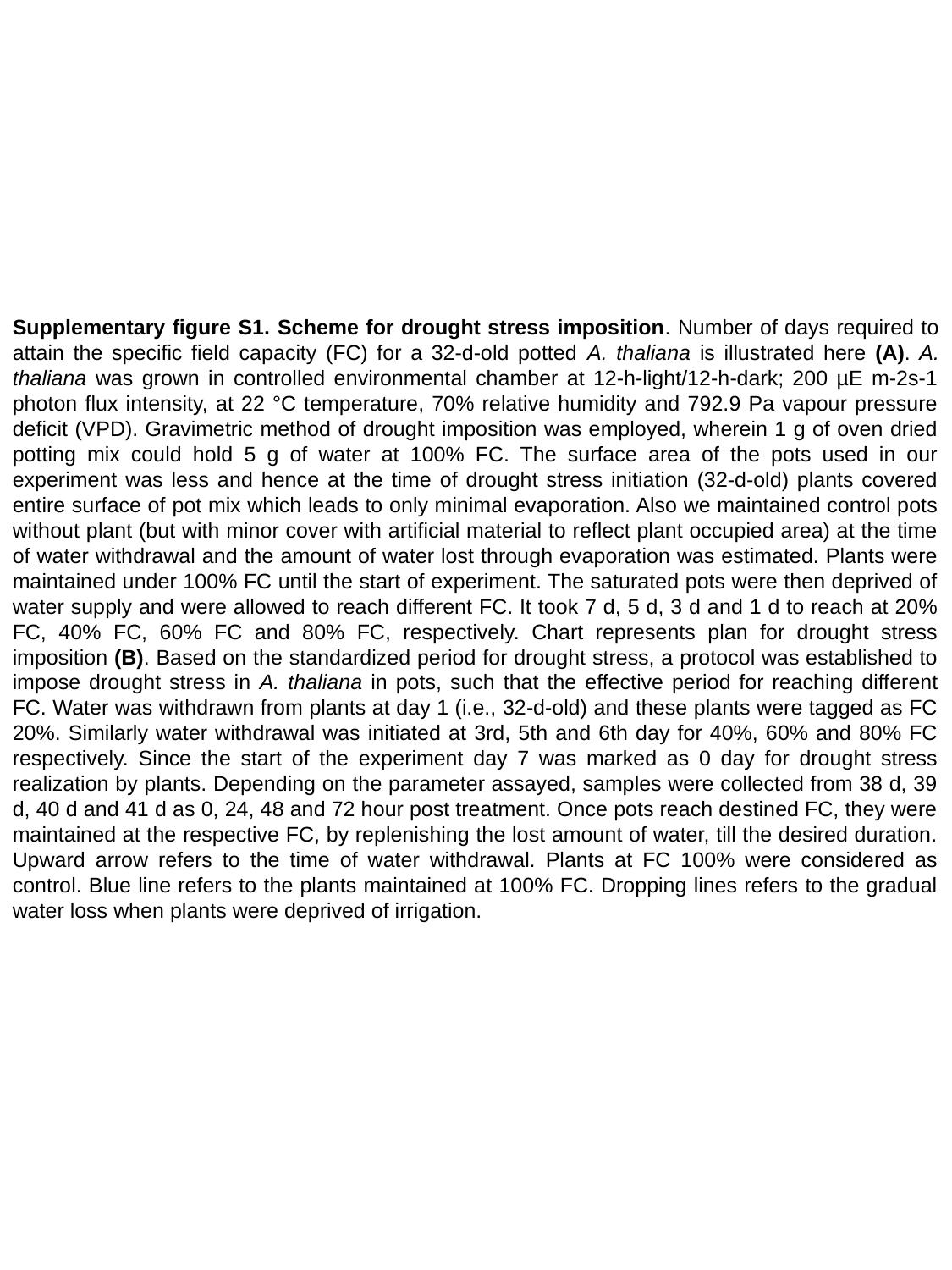

Supplementary figure S1. Scheme for drought stress imposition. Number of days required to attain the specific field capacity (FC) for a 32-d-old potted A. thaliana is illustrated here (A). A. thaliana was grown in controlled environmental chamber at 12-h-light/12-h-dark; 200 µE m-2s-1 photon flux intensity, at 22 °C temperature, 70% relative humidity and 792.9 Pa vapour pressure deficit (VPD). Gravimetric method of drought imposition was employed, wherein 1 g of oven dried potting mix could hold 5 g of water at 100% FC. The surface area of the pots used in our experiment was less and hence at the time of drought stress initiation (32-d-old) plants covered entire surface of pot mix which leads to only minimal evaporation. Also we maintained control pots without plant (but with minor cover with artificial material to reflect plant occupied area) at the time of water withdrawal and the amount of water lost through evaporation was estimated. Plants were maintained under 100% FC until the start of experiment. The saturated pots were then deprived of water supply and were allowed to reach different FC. It took 7 d, 5 d, 3 d and 1 d to reach at 20% FC, 40% FC, 60% FC and 80% FC, respectively. Chart represents plan for drought stress imposition (B). Based on the standardized period for drought stress, a protocol was established to impose drought stress in A. thaliana in pots, such that the effective period for reaching different FC. Water was withdrawn from plants at day 1 (i.e., 32-d-old) and these plants were tagged as FC 20%. Similarly water withdrawal was initiated at 3rd, 5th and 6th day for 40%, 60% and 80% FC respectively. Since the start of the experiment day 7 was marked as 0 day for drought stress realization by plants. Depending on the parameter assayed, samples were collected from 38 d, 39 d, 40 d and 41 d as 0, 24, 48 and 72 hour post treatment. Once pots reach destined FC, they were maintained at the respective FC, by replenishing the lost amount of water, till the desired duration. Upward arrow refers to the time of water withdrawal. Plants at FC 100% were considered as control. Blue line refers to the plants maintained at 100% FC. Dropping lines refers to the gradual water loss when plants were deprived of irrigation.

## Slide 3
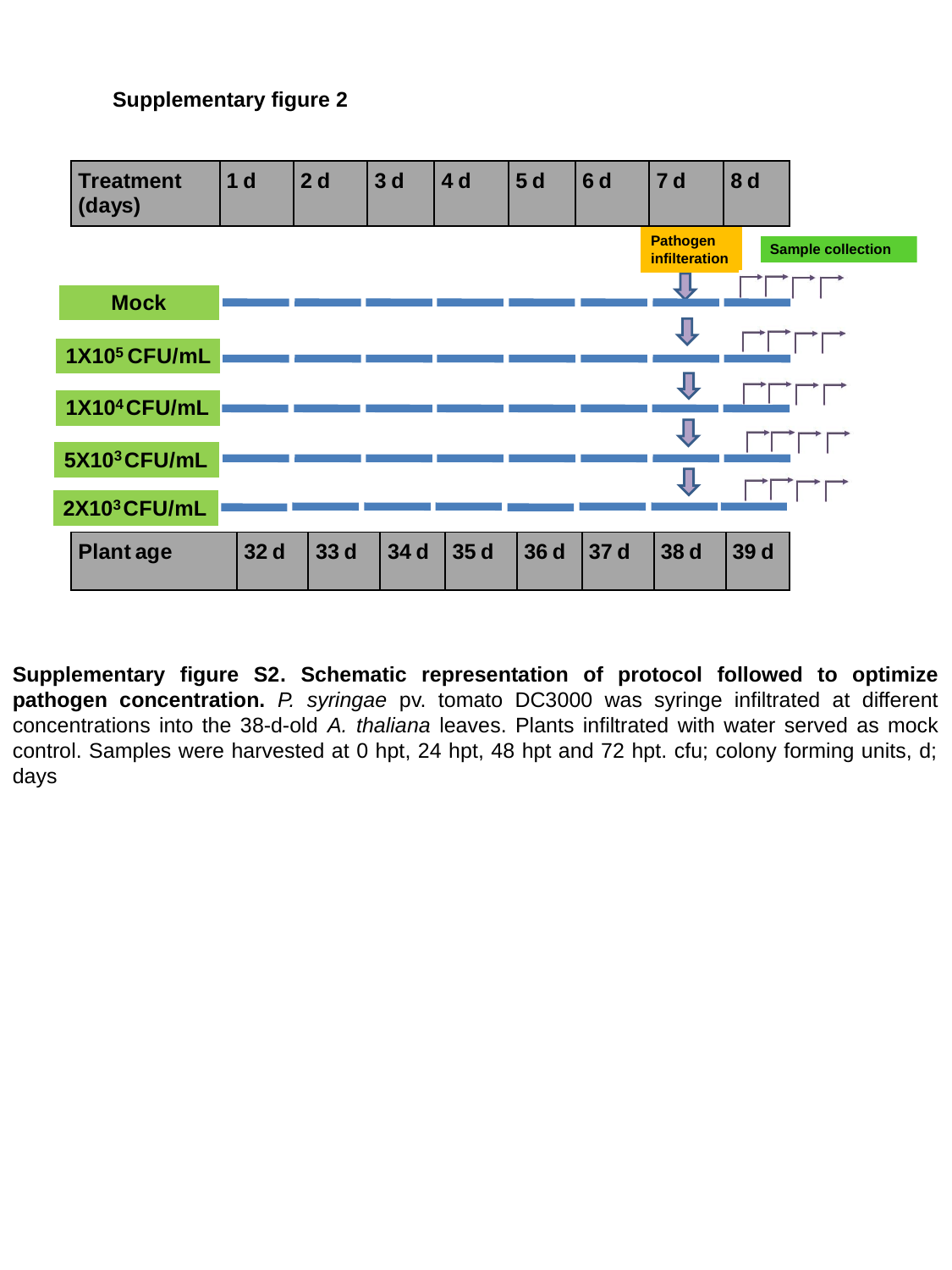

Supplementary figure 2
Supplementary figure S2. Schematic representation of protocol followed to optimize pathogen concentration. P. syringae pv. tomato DC3000 was syringe infiltrated at different concentrations into the 38-d-old A. thaliana leaves. Plants infiltrated with water served as mock control. Samples were harvested at 0 hpt, 24 hpt, 48 hpt and 72 hpt. cfu; colony forming units, d; days

## Slide 4
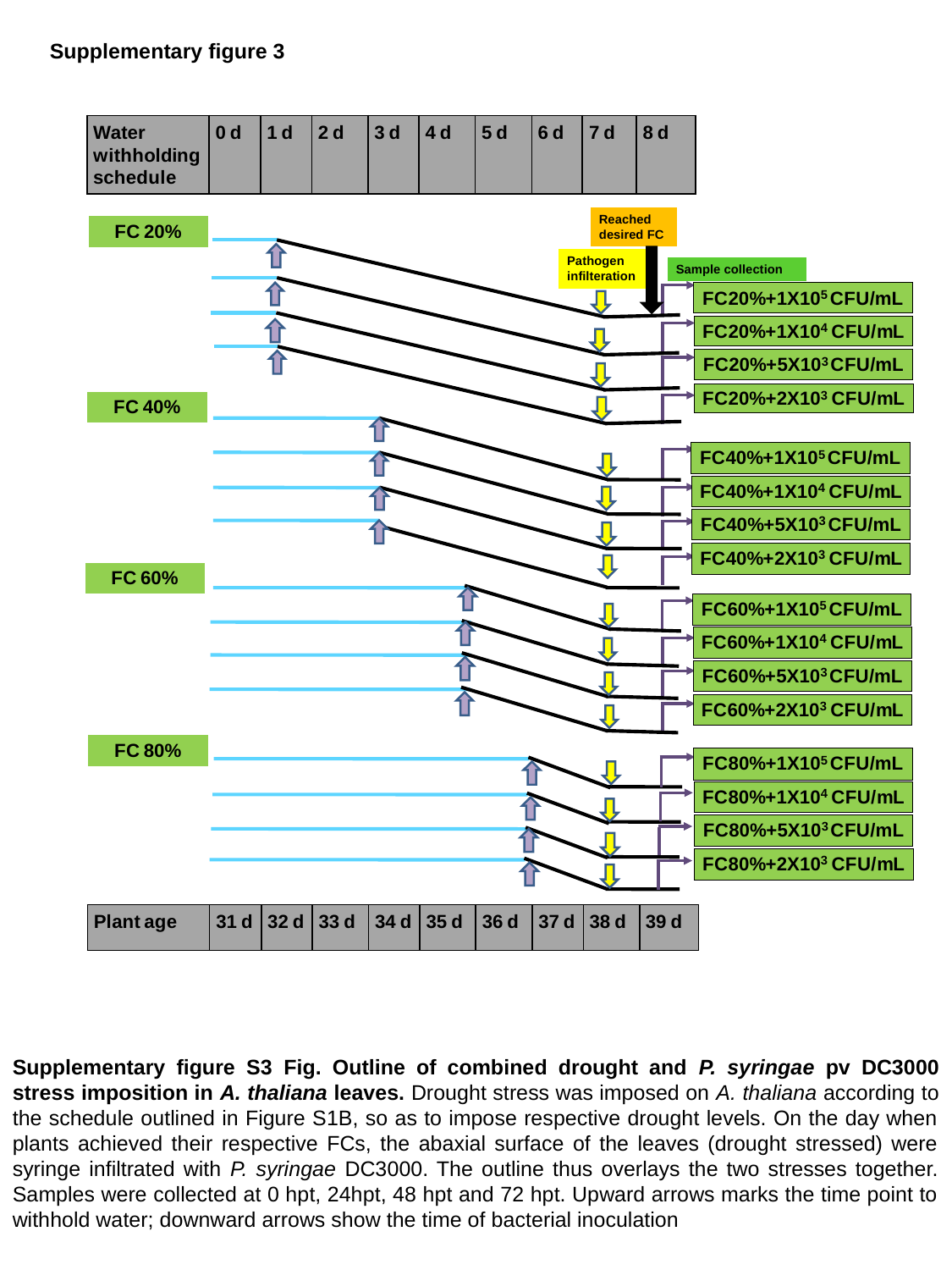

Supplementary figure 3
Supplementary figure S3 Fig. Outline of combined drought and P. syringae pv DC3000 stress imposition in A. thaliana leaves. Drought stress was imposed on A. thaliana according to the schedule outlined in Figure S1B, so as to impose respective drought levels. On the day when plants achieved their respective FCs, the abaxial surface of the leaves (drought stressed) were syringe infiltrated with P. syringae DC3000. The outline thus overlays the two stresses together. Samples were collected at 0 hpt, 24hpt, 48 hpt and 72 hpt. Upward arrows marks the time point to withhold water; downward arrows show the time of bacterial inoculation

## Slide 5
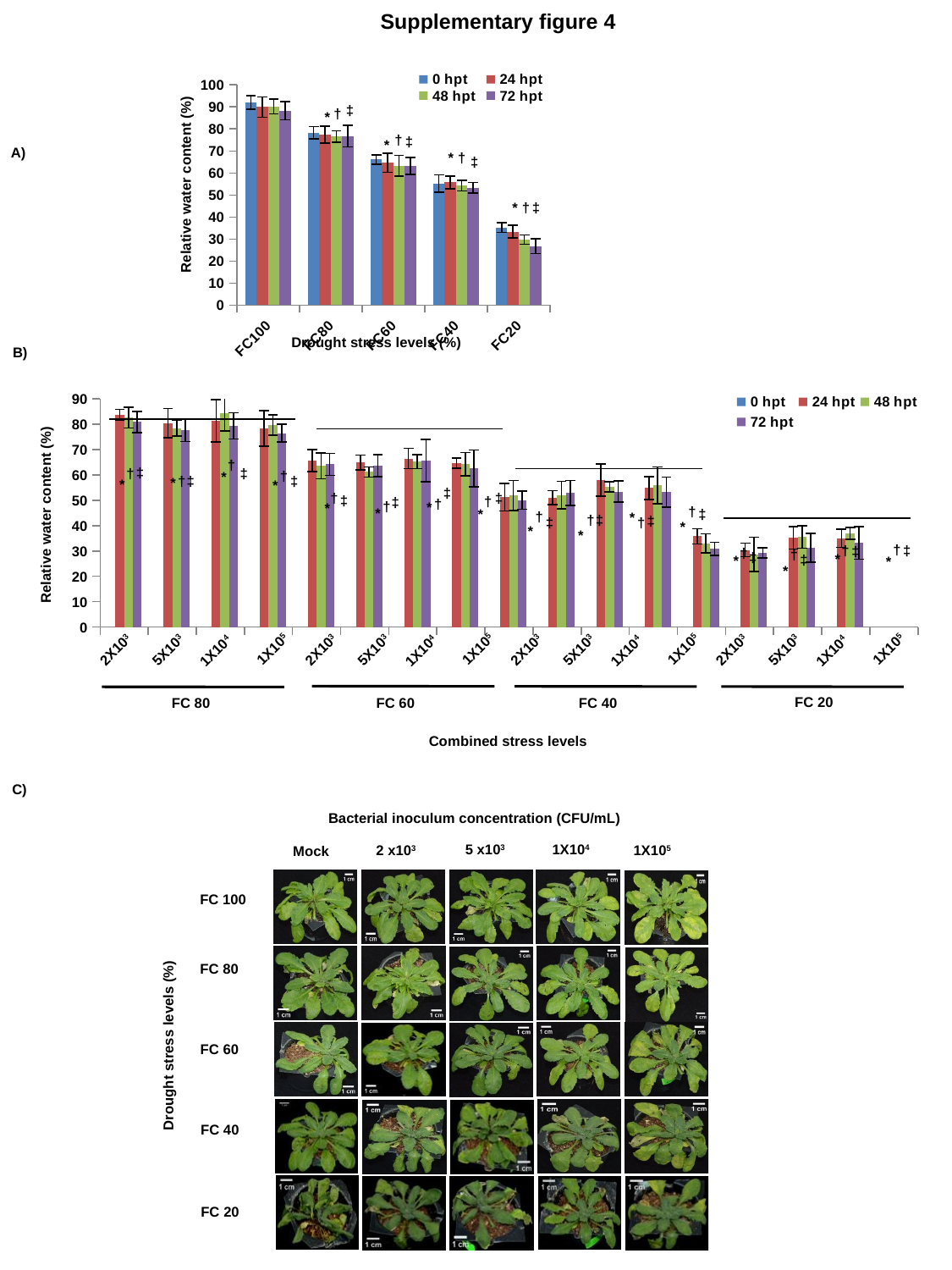

Supplementary figure 4
### Chart
| Category | 0 hpt | 24 hpt | 48 hpt | 72 hpt |
|---|---|---|---|---|
| FC100 | 92.00231099999998 | 89.90328379981604 | 90.22389015076882 | 88.28518282420418 |
| FC80 | 78.3044854881266 | 77.41994774875296 | 76.53576669449126 | 76.6963049058066 |
| FC60 | 66.11086999999999 | 64.62036976056318 | 63.29394340255601 | 63.18019689253551 |
| FC40 | 55.2055171638856 | 55.72375564034919 | 54.28571428571 | 53.22450000000001 |
| FC20 | 35.31212693795919 | 33.369580093118465 | 29.75206611570249 | 26.73913043478261 |Relative water content (%)
‡
†
*
†
‡
*
*
†
‡
*
†
‡
Drought stress levels (%)
A)
B)
†
‡
‡
†
†
*
†
‡
‡
*
*
*
‡
†
‡
‡
†
‡
†
†
*
*
†
*
‡
*
†
*
‡
†
‡
†
‡
*
*
*
†
†
‡
‡
†
†
‡
*
‡
*
*
*
Relative water content (%)
### Chart
| Category | 0 hpt | 24 hpt | 48 hpt | 72 hpt |
|---|---|---|---|---|
| FC80+2*10^4 | 113.29333 | 83.6963049058066 | 82.57364604012248 | 80.80591940772358 |
| FC80+5*10^4 | 111.04453000000002 | 80.419947748753 | 78.35431036784078 | 77.53576669449156 |
| FC80+10^6 | 115.81986143187066 | 81.28518282420455 | 84.36470818232377 | 79.29865268539156 |
| FC80+10^5 | 109.23854 | 78.26370520671038 | 79.69748535415708 | 76.4533940553505 |
| FC60+2*10^4 | 103.557656 | 65.5913911445453 | 63.53646203414426 | 64.18019689253528 |
| FC60+5*10^4 | 99.88790199999998 | 64.85957762233082 | 61.133256103 | 63.59139114454531 |
| FC60+10^5 | 107.4474953617811 | 66.44976516809994 | 65.2214423256103 | 65.6103 |
| FC60+10^6 | 102.54672897196262 | 64.62036976056318 | 64.18019689253528 | 62.5664675906937 |
| FC40+2*10^4 | 85.31012900000017 | 51.1648101435364 | 51.83488 | 50.00830000000001 |
| FC40+5*10^4 | 77.29120000000017 | 51.02792792792796 | 52.004657 | 52.8544 |
| FC40+10^5 | 80.97976391231029 | 57.9646017699114 | 55.23445400000001 | 53.3967 |
| FC40+10^6 | 83.40035799999998 | 54.800939613624394 | 55.900654 | 53.2276 |
| FC20+2*10^4 | 46.16113200000008 | 35.79211300000014 | 33.05443999999999 | 30.8301886792453 |
| FC20+5*10^4 | 53.5131 | 30.41037 | 28.65488000000005 | 29.311650000000043 |
| FC20+10^5 | 54.8956521739131 | 35.115640000000006 | 35.504348 | 31.3076 |
| FC20+10^6 | 49.17971400000001 | 34.98168498168486 | 36.8875228 | 33.223750000000095 |1X105
1X105
1X105
1X105
2X103
2X103
2X103
2X103
5X103
5X103
5X103
5X103
1X104
1X104
1X104
1X104
FC 20
FC 40
FC 60
FC 80
Combined stress levels
C)
Bacterial inoculum concentration (CFU/mL)
FC 100
FC 80
Drought stress levels (%)
FC 60
FC 40
FC 20
5 x10^3^
1X104
2 x10^3^
1X105
Mock

## Slide 6
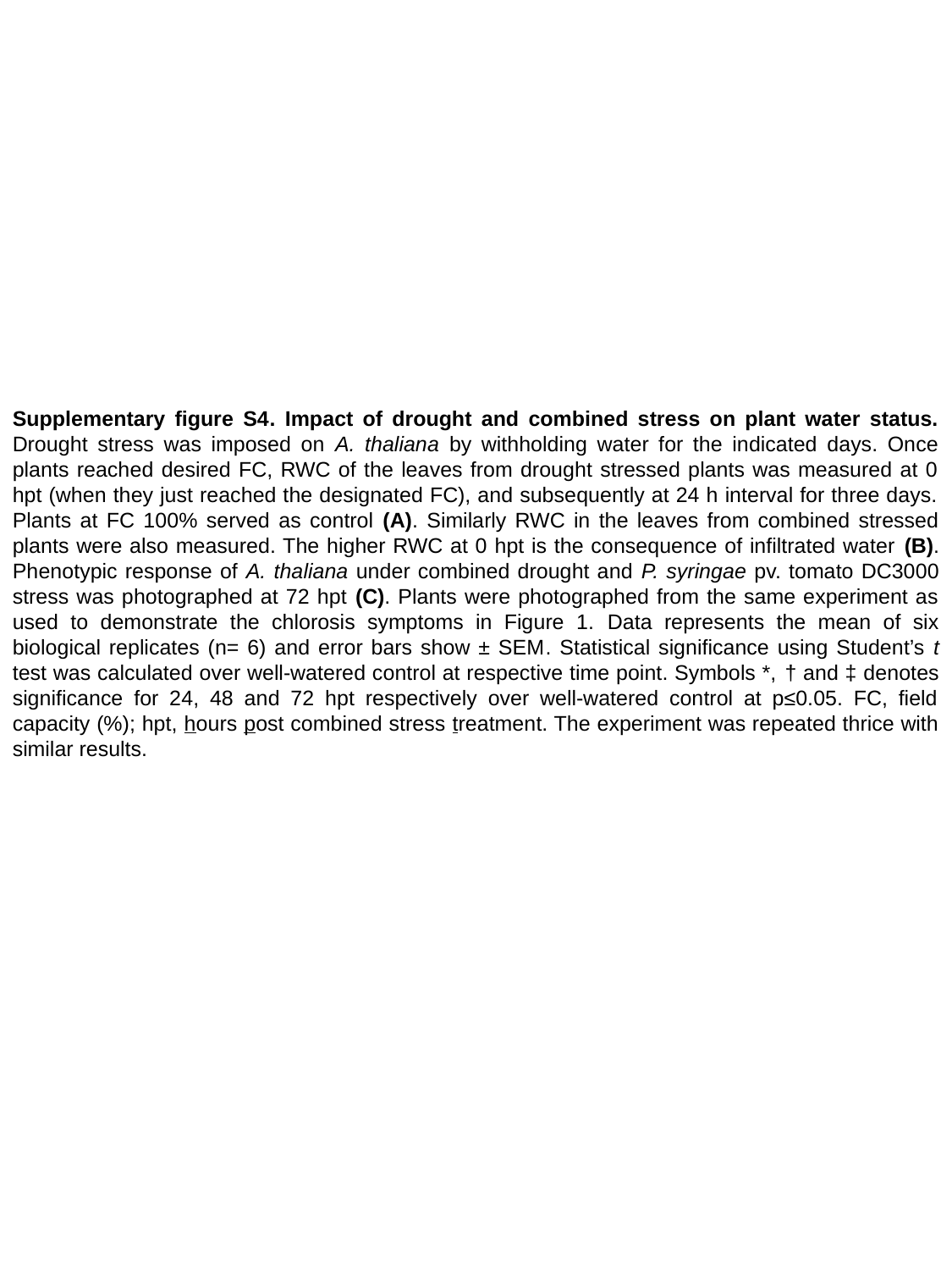

Supplementary figure S4. Impact of drought and combined stress on plant water status. Drought stress was imposed on A. thaliana by withholding water for the indicated days. Once plants reached desired FC, RWC of the leaves from drought stressed plants was measured at 0 hpt (when they just reached the designated FC), and subsequently at 24 h interval for three days. Plants at FC 100% served as control (A). Similarly RWC in the leaves from combined stressed plants were also measured. The higher RWC at 0 hpt is the consequence of infiltrated water (B). Phenotypic response of A. thaliana under combined drought and P. syringae pv. tomato DC3000 stress was photographed at 72 hpt (C). Plants were photographed from the same experiment as used to demonstrate the chlorosis symptoms in Figure 1. Data represents the mean of six biological replicates (n= 6) and error bars show ± SEM. Statistical significance using Student’s t test was calculated over well-watered control at respective time point. Symbols *, † and ‡ denotes significance for 24, 48 and 72 hpt respectively over well-watered control at p≤0.05. FC, field capacity (%); hpt, hours post combined stress treatment. The experiment was repeated thrice with similar results.

## Slide 7
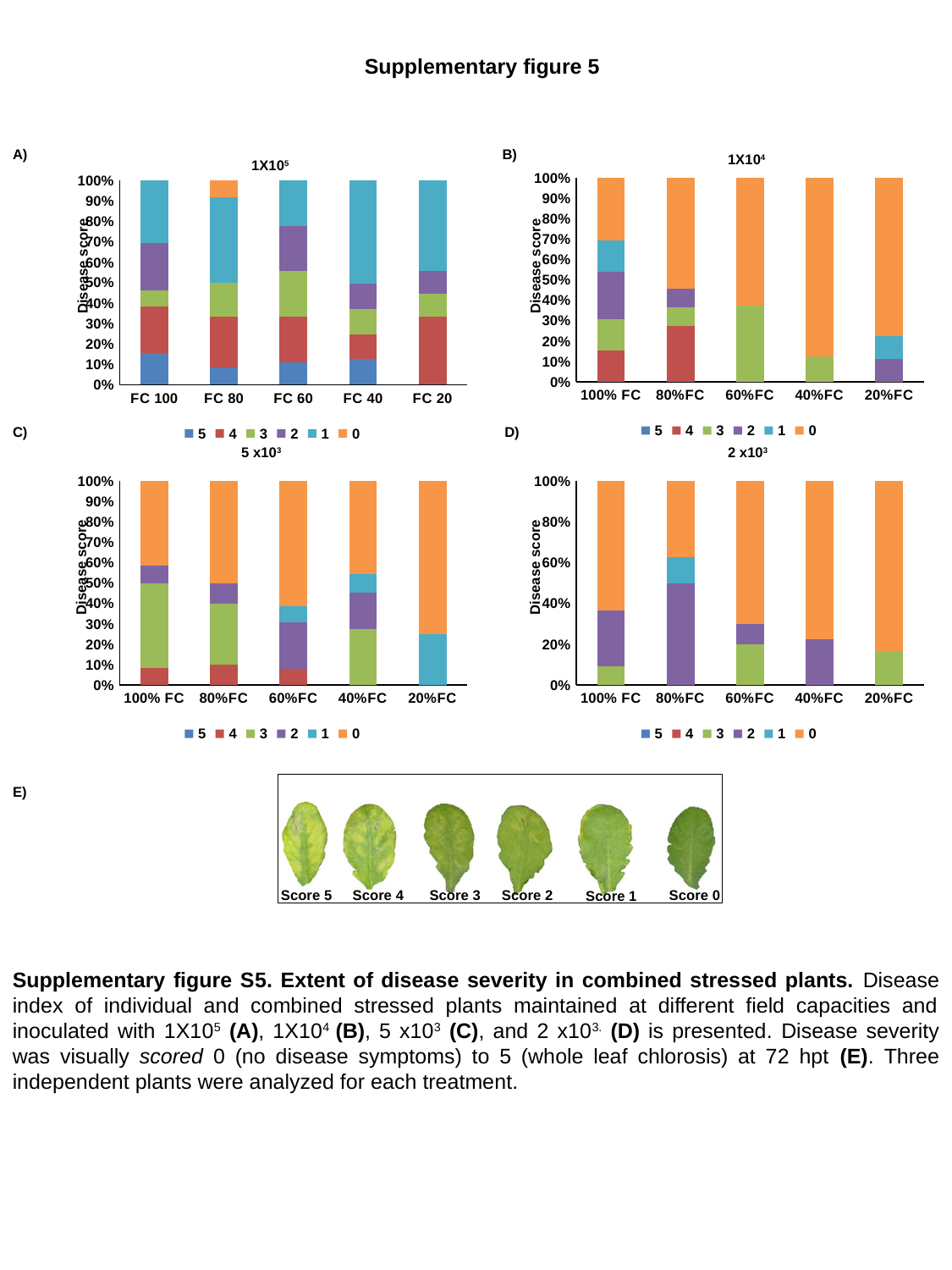

Supplementary figure 5
A)
B)
1X104
### Chart
| Category | 5 | 4 | 3 | 2 | 1 | 0 |
|---|---|---|---|---|---|---|
| 100% FC | 0.0 | 15.384615384615385 | 15.384615384615385 | 23.07692307692304 | 15.384615384615385 | 30.76923076923077 |
| 80%FC | 0.0 | 27.27272727272718 | 9.0909090909091 | 9.0909090909091 | 0.0 | 54.54545454545455 |
| 60%FC | 0.0 | 0.0 | 37.5 | 0.0 | 0.0 | 62.5 |
| 40%FC | 0.0 | 0.0 | 12.5 | 0.0 | 0.0 | 87.5 |
| 20%FC | 0.0 | 0.0 | 0.0 | 11.111111111111091 | 11.111111111111091 | 77.77777777777766 |1X105
### Chart
| Category | 5 | 4 | 3 | 2 | 1 | 0 |
|---|---|---|---|---|---|---|
| FC 100 | 15.384615384615385 | 23.07692307692304 | 7.6923076923076925 | 23.07692307692304 | 30.76923076923077 | 0.0 |
| FC 80 | 8.333333333333336 | 25.0 | 16.666666666666668 | 0.0 | 41.66666666666655 | 8.333333333333336 |
| FC 60 | 11.111111111111091 | 22.222222222222175 | 22.222222222222175 | 22.222222222222175 | 22.222222222222175 | 0.0 |
| FC 40 | 11.111111111111091 | 10.526315789473669 | 11.111111111111091 | 11.111111111111091 | 44.44444444444433 | 0.0 |
| FC 20 | 0.0 | 33.333333333333336 | 11.111111111111091 | 11.111111111111091 | 44.44444444444433 | 0.0 |Disease score
Disease score
C)
D)
5 x10^3^
### Chart
| Category | 5 | 4 | 3 | 2 | 1 | 0 |
|---|---|---|---|---|---|---|
| 100% FC | 0.0 | 8.333333333333336 | 41.66666666666655 | 8.333333333333336 | 0.0 | 41.66666666666655 |
| 80%FC | 0.0 | 10.0 | 30.0 | 10.0 | 0.0 | 50.0 |
| 60%FC | 0.0 | 9.0909090909091 | 0.0 | 27.27272727272718 | 9.0909090909091 | 72.72727272727273 |
| 40%FC | 0.0 | 0.0 | 27.27272727272718 | 18.18181818181822 | 9.0909090909091 | 45.45454545454537 |
| 20%FC | 0.0 | 0.0 | 0.0 | 0.0 | 25.0 | 75.0 |2 x10^3^
### Chart
| Category | 5 | 4 | 3 | 2 | 1 | 0 |
|---|---|---|---|---|---|---|
| 100% FC | 0.0 | 0.0 | 9.0909090909091 | 27.27272727272718 | 0.0 | 63.636363636363626 |
| 80%FC | 0.0 | 0.0 | 0.0 | 50.0 | 12.5 | 37.5 |
| 60%FC | 0.0 | 0.0 | 20.0 | 10.0 | 0.0 | 70.0 |
| 40%FC | 0.0 | 0.0 | 0.0 | 22.222222222222175 | 0.0 | 77.77777777777766 |
| 20%FC | 0.0 | 0.0 | 16.666666666666668 | 0.0 | 0.0 | 83.33333333333326 |Disease score
Disease score
Score 5
Score 3
Score 0
Score 2
Score 4
Score 1
E)
Supplementary figure S5. Extent of disease severity in combined stressed plants. Disease index of individual and combined stressed plants maintained at different field capacities and inoculated with 1X105 (A), 1X104 (B), 5 x10^3^ (C), and 2 x10^3^. (D) is presented. Disease severity was visually scored 0 (no disease symptoms) to 5 (whole leaf chlorosis) at 72 hpt (E). Three independent plants were analyzed for each treatment.

## Slide 8
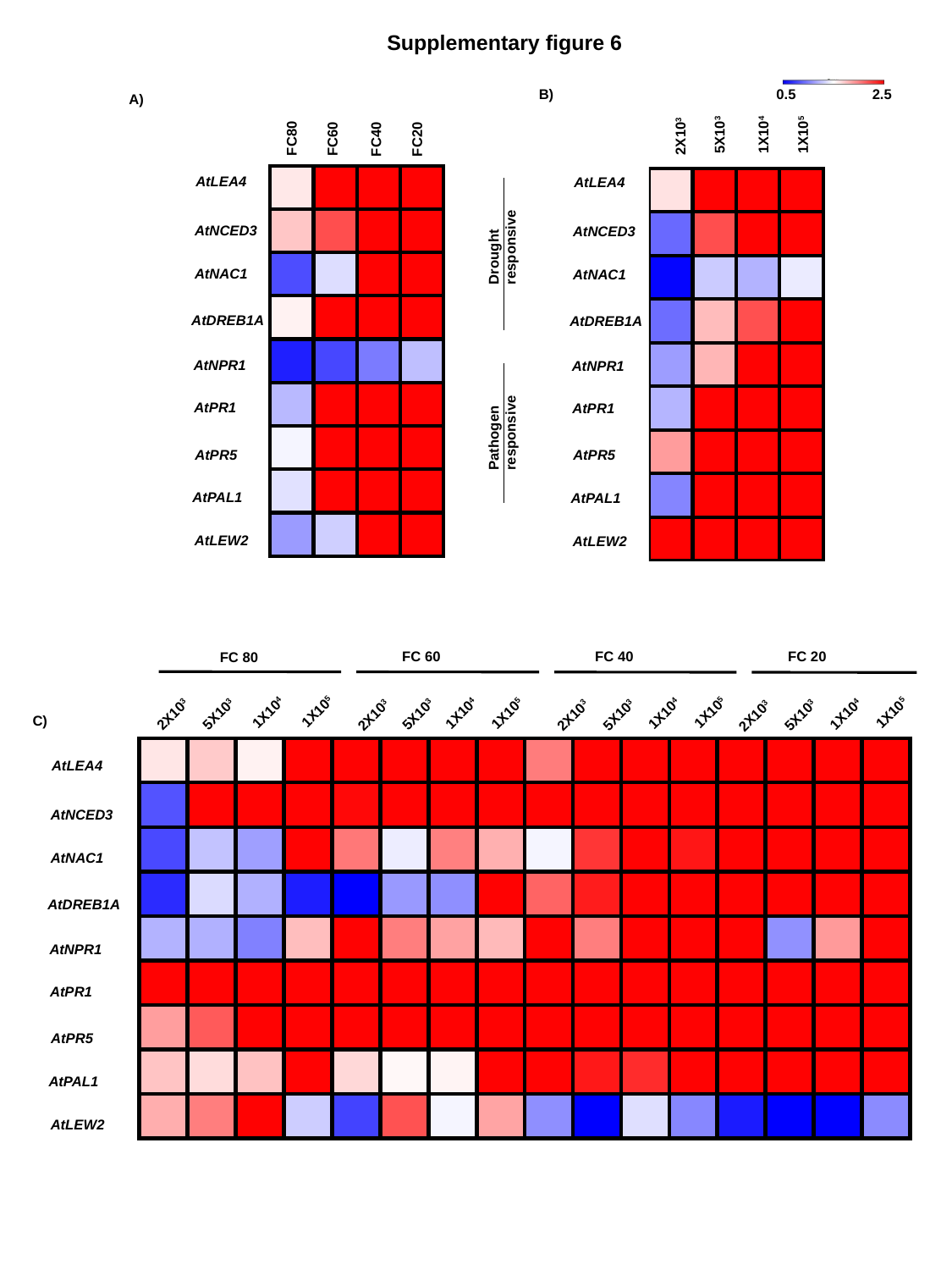

Supplementary figure 6
0.5 2.5
B)
A)
5X103
1X104
1X105
2X103
AtLEA4
AtNCED3
AtNAC1
AtDREB1A
AtNPR1
AtPR1
AtPR5
AtPAL1
AtLEW2
FC80
FC60
FC40
FC20
AtLEA4
AtNCED3
AtNAC1
AtDREB1A
AtNPR1
AtPR1
AtPR5
AtPAL1
AtLEW2
Drought responsive
Pathogen responsive
FC 20
FC 40
FC 60
FC 80
1X105
1X105
1X104
1X105
1X104
1X105
1X104
5X103
1X104
5X103
2X103
5X103
2X103
5X103
2X103
2X103
AtLEA4
AtNCED3
AtNAC1
AtDREB1A
AtPR1
AtPR5
AtPAL1
AtLEW2
AtNPR1
C)

## Slide 9
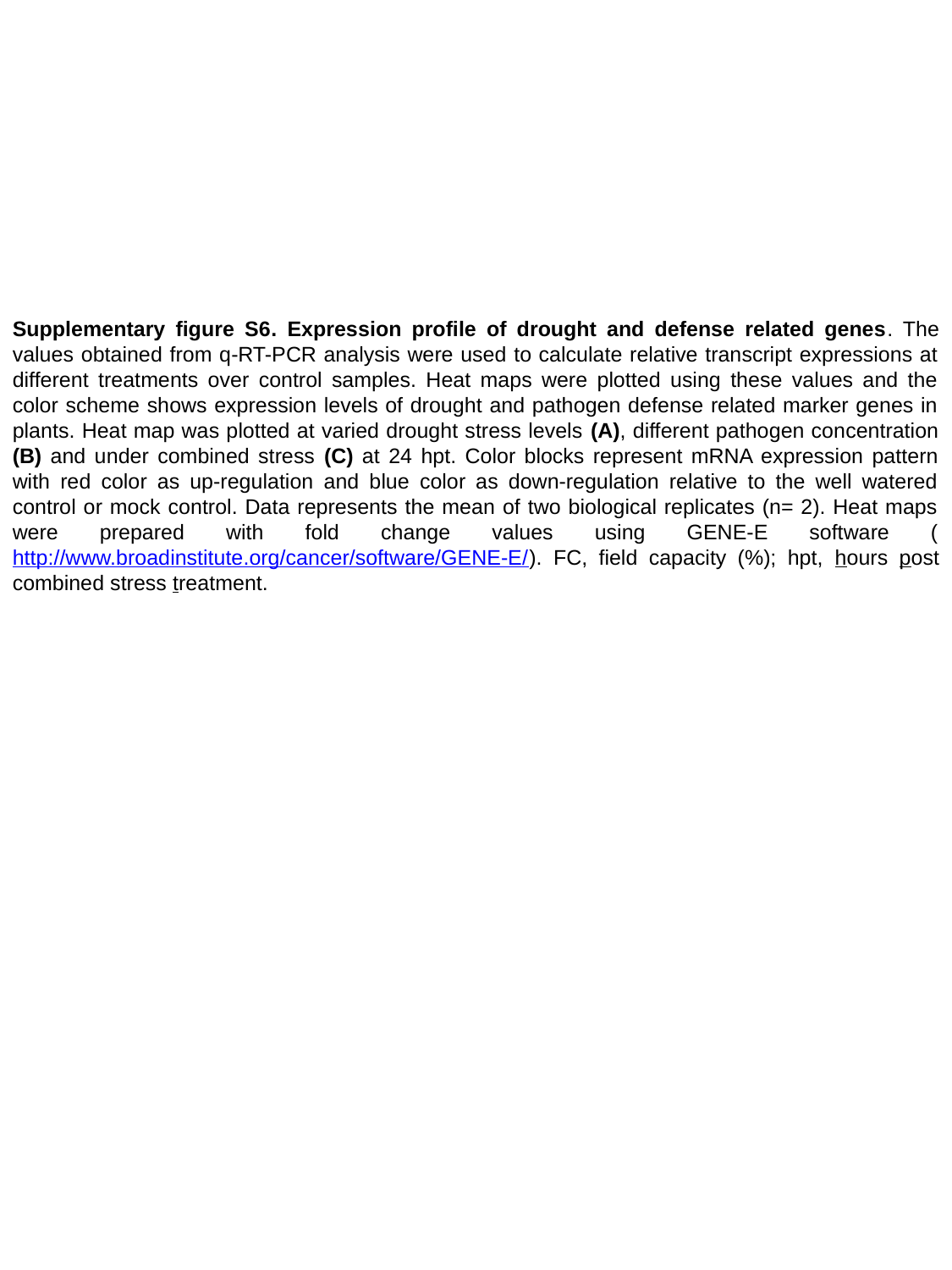

Supplementary figure S6. Expression profile of drought and defense related genes. The values obtained from q-RT-PCR analysis were used to calculate relative transcript expressions at different treatments over control samples. Heat maps were plotted using these values and the color scheme shows expression levels of drought and pathogen defense related marker genes in plants. Heat map was plotted at varied drought stress levels (A), different pathogen concentration (B) and under combined stress (C) at 24 hpt. Color blocks represent mRNA expression pattern with red color as up-regulation and blue color as down-regulation relative to the well watered control or mock control. Data represents the mean of two biological replicates (n= 2). Heat maps were prepared with fold change values using GENE-E software (http://www.broadinstitute.org/cancer/software/GENE-E/). FC, field capacity (%); hpt, hours post combined stress treatment.

## Slide 10
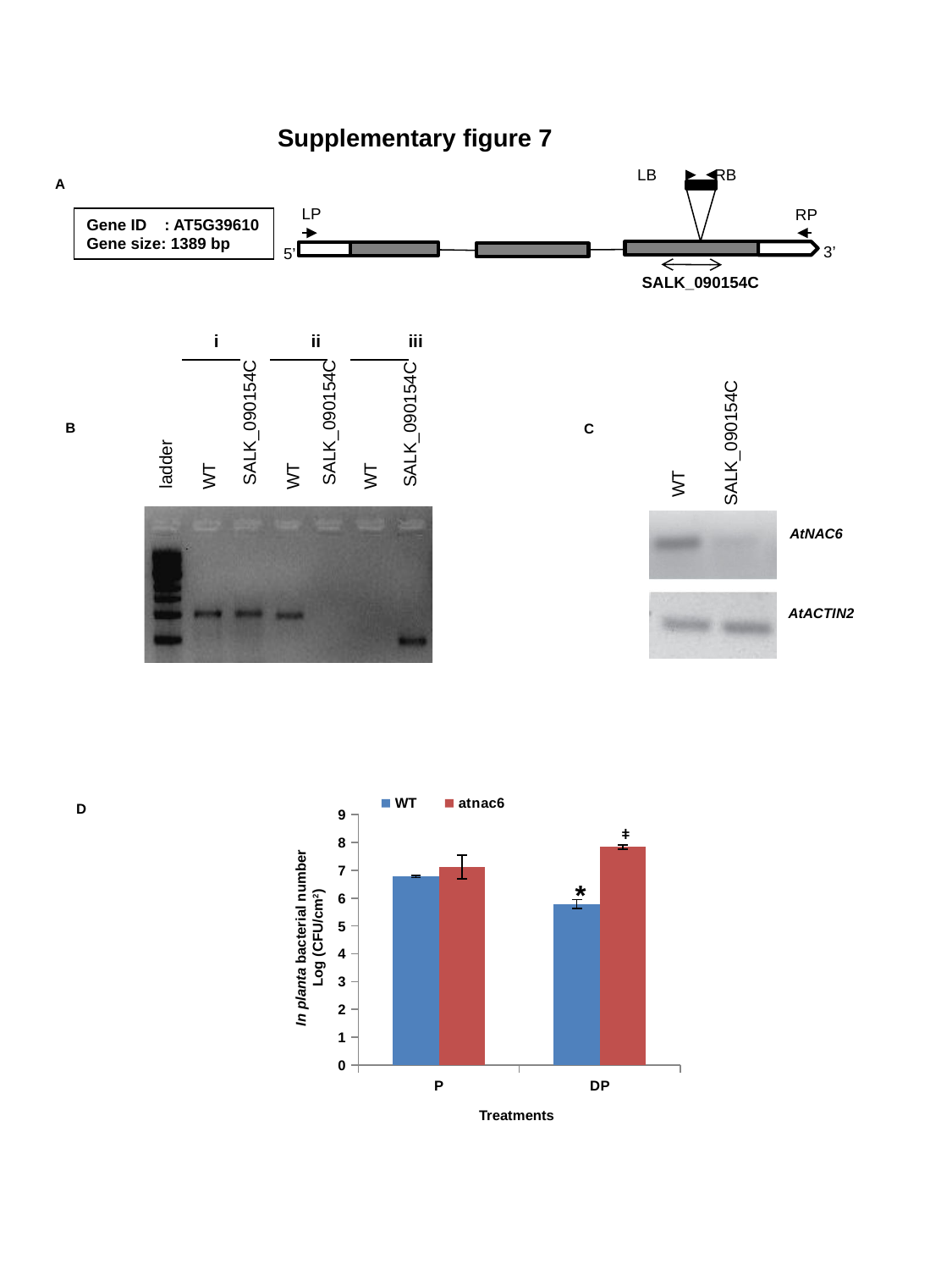

Supplementary figure 7
LB RB
LP
RP
Gene ID : AT5G39610
Gene size: 1389 bp
3’
5’
SALK_090154C
A
i ii iii
SALK_090154C
SALK_090154C
SALK_090154C
ladder
WT
WT
WT
SALK_090154C
WT
AtNAC6
AtACTIN2
B
C
### Chart
| Category | WT | atnac6 |
|---|---|---|
| P | 6.783831686838131 | 7.115106220836844 |
| DP | 5.78828843150289 | 7.84036130532533 |In planta bacterial number
Log (CFU/cm2)
Treatments
*
D
ǂ

## Slide 11
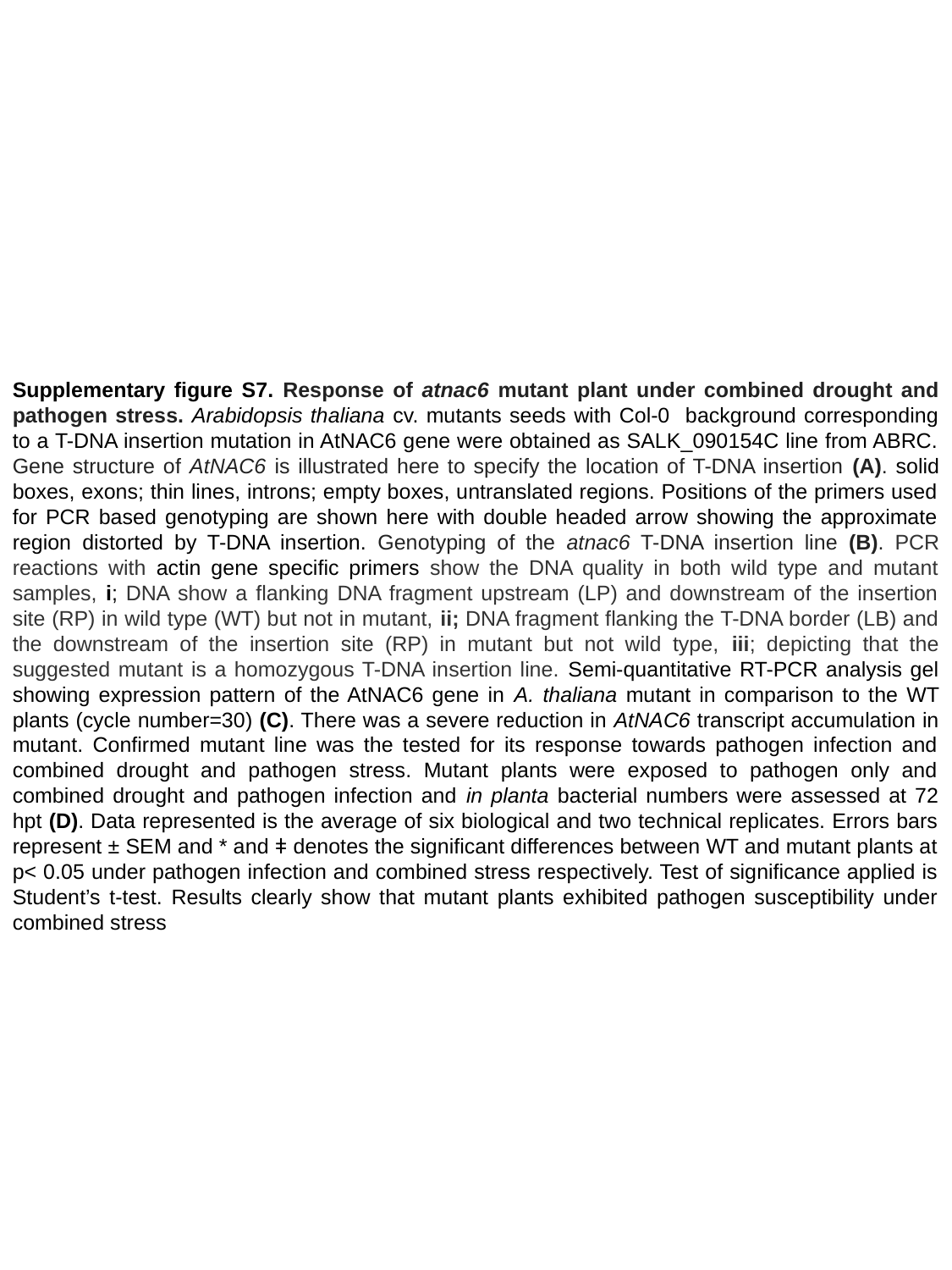

Supplementary figure S7. Response of atnac6 mutant plant under combined drought and pathogen stress. Arabidopsis thaliana cv. mutants seeds with Col-0 background corresponding to a T-DNA insertion mutation in AtNAC6 gene were obtained as SALK_090154C line from ABRC. Gene structure of AtNAC6 is illustrated here to specify the location of T-DNA insertion (A). solid boxes, exons; thin lines, introns; empty boxes, untranslated regions. Positions of the primers used for PCR based genotyping are shown here with double headed arrow showing the approximate region distorted by T-DNA insertion. Genotyping of the atnac6 T-DNA insertion line (B). PCR reactions with actin gene specific primers show the DNA quality in both wild type and mutant samples, i; DNA show a flanking DNA fragment upstream (LP) and downstream of the insertion site (RP) in wild type (WT) but not in mutant, ii; DNA fragment flanking the T-DNA border (LB) and the downstream of the insertion site (RP) in mutant but not wild type, iii; depicting that the suggested mutant is a homozygous T-DNA insertion line. Semi-quantitative RT-PCR analysis gel showing expression pattern of the AtNAC6 gene in A. thaliana mutant in comparison to the WT plants (cycle number=30) (C). There was a severe reduction in AtNAC6 transcript accumulation in mutant. Confirmed mutant line was the tested for its response towards pathogen infection and combined drought and pathogen stress. Mutant plants were exposed to pathogen only and combined drought and pathogen infection and in planta bacterial numbers were assessed at 72 hpt (D). Data represented is the average of six biological and two technical replicates. Errors bars represent ± SEM and * and ǂ denotes the significant differences between WT and mutant plants at p< 0.05 under pathogen infection and combined stress respectively. Test of significance applied is Student’s t-test. Results clearly show that mutant plants exhibited pathogen susceptibility under combined stress

## Slide 12
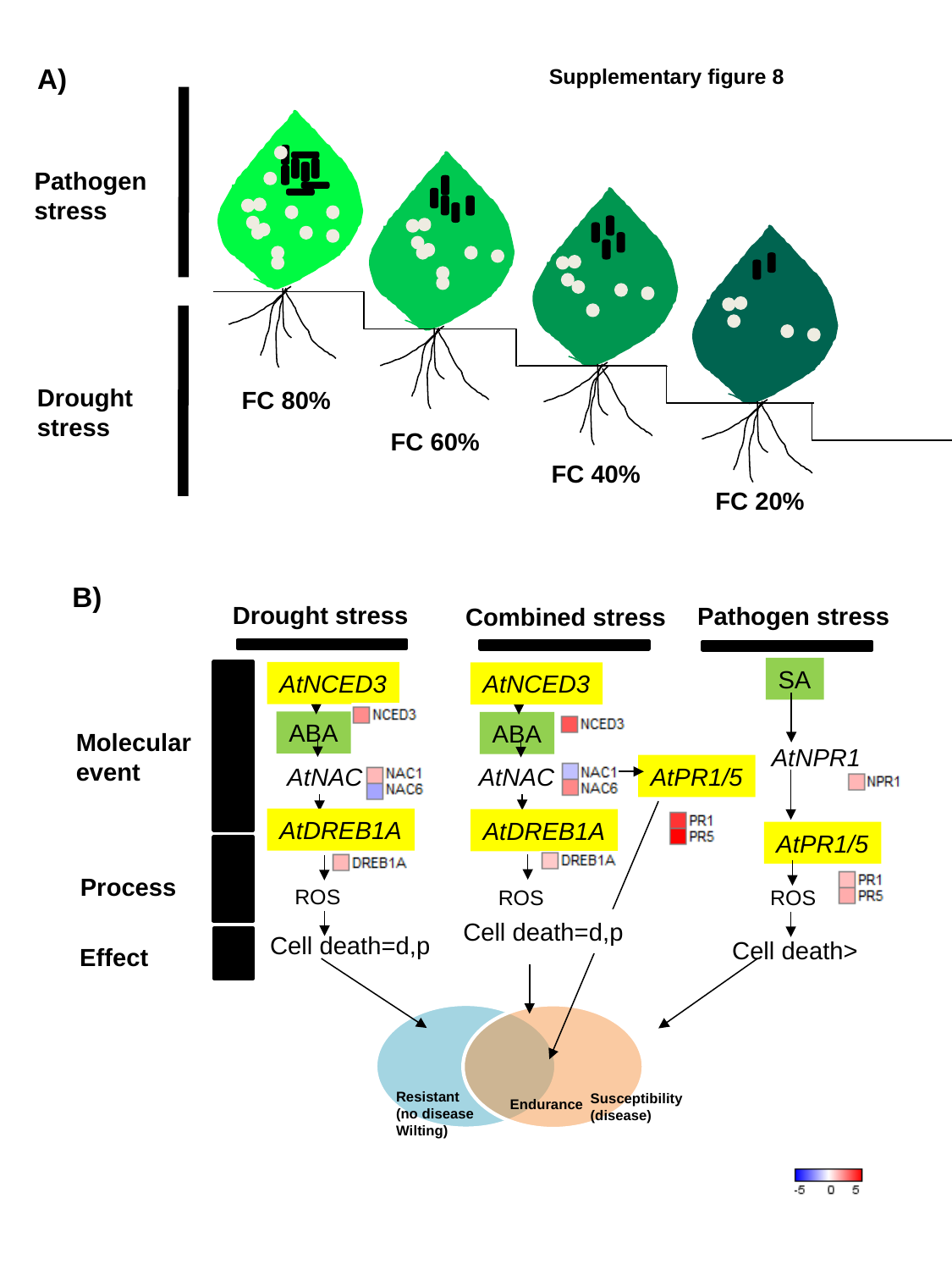

A)
Supplementary figure 8
Pathogen stress
Drought stress
FC 80%
FC 60%
FC 40%
FC 20%
B)
Drought stress
Pathogen stress
Combined stress
SA
AtNPR1
AtPR1/5
Cell death>
AtNCED3
ABA
AtNAC
AtDREB1A
Cell death=d,p
ROS
AtNCED3
ABA
AtNAC
AtDREB1A
AtPR1/5
ROS
ROS
Molecular event
Process
Effect
Resistant
(no disease
Wilting)
Susceptibility
(disease)
Endurance
Cell death=d,p

## Slide 13
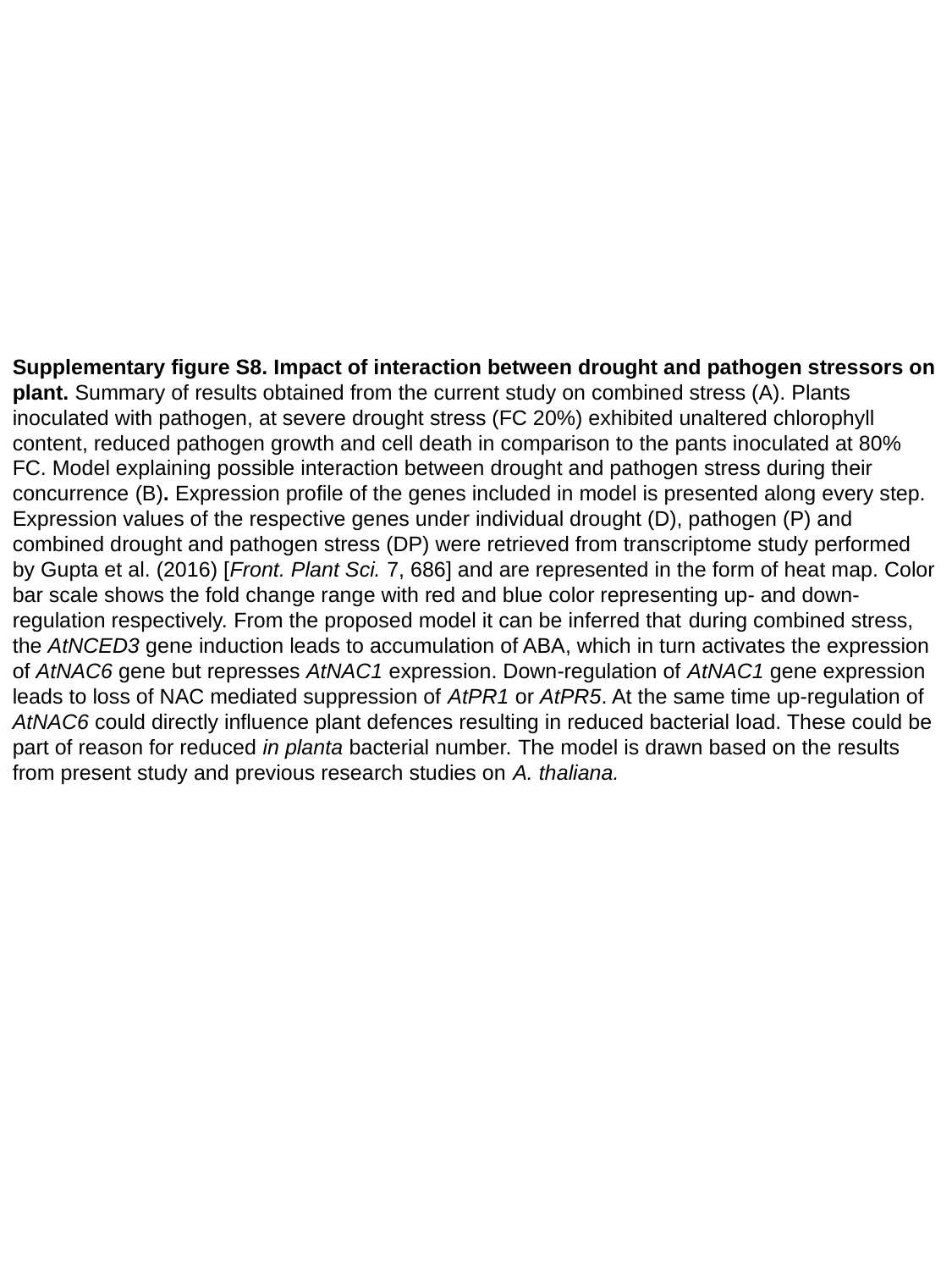

Supplementary figure S8. Impact of interaction between drought and pathogen stressors on plant. Summary of results obtained from the current study on combined stress (A). Plants inoculated with pathogen, at severe drought stress (FC 20%) exhibited unaltered chlorophyll content, reduced pathogen growth and cell death in comparison to the pants inoculated at 80% FC. Model explaining possible interaction between drought and pathogen stress during their concurrence (B). Expression profile of the genes included in model is presented along every step. Expression values of the respective genes under individual drought (D), pathogen (P) and combined drought and pathogen stress (DP) were retrieved from transcriptome study performed by Gupta et al. (2016) [Front. Plant Sci. 7, 686] and are represented in the form of heat map. Color bar scale shows the fold change range with red and blue color representing up- and down-regulation respectively. From the proposed model it can be inferred that during combined stress, the AtNCED3 gene induction leads to accumulation of ABA, which in turn activates the expression of AtNAC6 gene but represses AtNAC1 expression. Down-regulation of AtNAC1 gene expression leads to loss of NAC mediated suppression of AtPR1 or AtPR5. At the same time up-regulation of AtNAC6 could directly influence plant defences resulting in reduced bacterial load. These could be part of reason for reduced in planta bacterial number. The model is drawn based on the results from present study and previous research studies on A. thaliana.
