# Supplementary material for: Drought Stress Predominantly Endures Arabidopsis thaliana to Pseudomonas syringae Infection
Source: Front Plant Sci. 2016 Jun 7;7:808. doi: 10.3389/fpls.2016.00808 (PMC4894909; doi:10.3389/fpls.2016.00808)
Supplement: Supplementary file 6 [file DataSheet1.DOC]

**File S2. Detailed protocol for combined drought and pathogen infection in Arabidopsis.**

Supplementary material methods: In order to achieve desired drought stress levels, potting mixture used for growing *A. thaliana* plants were handled according to the steps described below.

1. Autoclaved and completely dried vermiculite (Keltech Energies pvt. Ltd., Maharashtra, India) and agropeat (Prakruthi Agri Cocopeat Industry, Karnataka, India) was taken in a 3:1 (vol/vol) ratio. The potting mix was evenly mixed and was homogenous.
2. Small pots (5 cm dia) were used in the experiment. This pot mix volume facilitated adequate water holding just enough for the plant and the 32 d old plants were able to extract water through transpiration and dry up the pot mixture in least number of days to reach desired soil water status (field capacity). Pot volume was adequate enough for proper root growth.
3. The potting mix had bulk density of 0.177gm/cm3. Since the potting mix tends to be fluffy, pre weighed potting mix was filled in the pot by pounding the pot against the ground gently in order to settle the mix in the pot. This was done to make sure enough number of air spaces in the potting spaces.
4. Filled pot mix was saturated with half strength Hoglands’ solution (bottom irrigated) and one seed per pot was sown. Stratification was done for 48 h at 4 C to break the seed dormancy and thereafter plants were transferred to the growth chambers. Pots were kept in trays.
5. Plants of same sizes were selected at the start of the stress regime, in order to avoid the different rate of transpiration or differences in demand for water uptake from pot mixture, which may otherwise alter the water status in plants.
6. During drought stress imposition, pots were taken out from trays and were kept on the chamber floor. The air flow from the chamber floor, through the bottom of the pots helped in faster imposition of drought stress.
7. The water holding capacity of the potting mix was 5.11 g. Hence for the treatments involving drought stress, the controls were maintained at FC 100%. Drought stress imposition involves dry down method to bring plants at specific FC (80%, 60%, 40% and 20%). In this protocol plants reach at different FC at different times and will be maintained continuously at respective FC’s until the last set reaching 20% FC (Ramegowda et al 2013). In such case plants experience drought stress levels for different durations which may change the inference. Thus as an alternative we started water withdrawal for different FC at different days, when they attain their respective FC at the same day. We thus achieved the drought stress levels without altering the stress duration.
8. In order to avoid the recovery cycle during water replenishment, water was added away from the plant roots.
9. Evaporative water loss from the pot mix was minimum at the time of stress imposition.

**Reference**

Ramegowda V, Senthil-Kumar M, Ishiga Y, Kaundal A, Udayakumar M, Mysore KS (2013) Drought stress acclimation imparts tolerance to *Sclerotinia sclerotiorum* and *Pseudomonas syringae* in *Nicotiana benthamiana*. Int J Mol Sci, 14: 9497–9513.
